# Rational design of lipid-based nanoparticles for targeted anticancer therapies

**DOI:** 10.1007/s13346-026-02110-z

**Published:** 2026-04-09

**Authors:** María Arenas-Moreira, Alberto Ocaña, Carlos Alonso-Moreno, Iván Bravo

**Affiliations:** 1 Departamento de Química Inorgánica, Orgánica y Bioquímica, Facultad de Farmacia-Centro de Innovación en Química Avanzada (ORFEO-CINQA), Unidad nanoDrug, Universidad de Castilla-La Mancha, Albacete, 02008 Spain; 2 Experimental Therapeutics in Cancer Unit, Instituto de Investigación Sanitaria San Carlos (IdISSC), Madrid, Spain; 3 START Madrid-Fundación Jiménez Díaz (FJD) Early Phase Program, Fundación Jiménez Díaz Hospital, Madrid, Spain; 4 Departamento de Química Física, Facultad de Farmacia, Unidad nanoDrug, Universidad de Castilla-La Mancha, Albacete, 02008 Spain

**Keywords:** Lipid-based nanoparticles, Targeted therapy, Nanomedicine, Cancer treatment, Clinical translation

## Abstract

Targeted anticancer therapies, including monoclonal antibodies, antibody–drug conjugates, siRNA, small-molecule inhibitors, and PROTACs, offer precise treatments but face severe pharmacokinetic and biological barriers, such as poor bioavailability, limited tumor penetration, and off-target toxicity. While first-generation lipid-based nanoparticles (LBNPs) successfully utilized the enhanced permeability and retention (EPR) effect, relying exclusively on passive targeting is insufficient due to tumor heterogeneity. Therefore, this review provides an integrative analysis focused on the rational design of LBNPs. We systematically explore how the distinct structural complexities and biological barriers of each therapeutic modality strictly dictate specific LBNP design rules. The optimization of various nanocarriers—including liposomes, solid-lipid nanoparticles, and nanostructured lipid carriers—is discussed through customized lipid compositions, surface functionalization for active targeting, and the incorporation of ionizable lipids to overcome intracellular barriers like endosomal entrapment. Furthermore, these structural designs are correlated with optimal administration routes, and the impact of formulation methods is evaluated by contrasting traditional emulsification with advanced continuous platforms like microfluidics and supercritical fluid technology. Finally, the clinical landscape and translational challenges of approved and experimental nanomedicines are assessed. We conclude that the transition from bench to bedside is currently hindered less by preclinical efficacy and more by manufacturing and regulatory bottlenecks. Overcoming chemistry, manufacturing, and controls (CMC) challenges, ensuring robust industrial scalability, and establishing harmonized regulatory frameworks are critical priorities for the future clinical success of targeted nanomedicines.

**Graphical Abstract:**

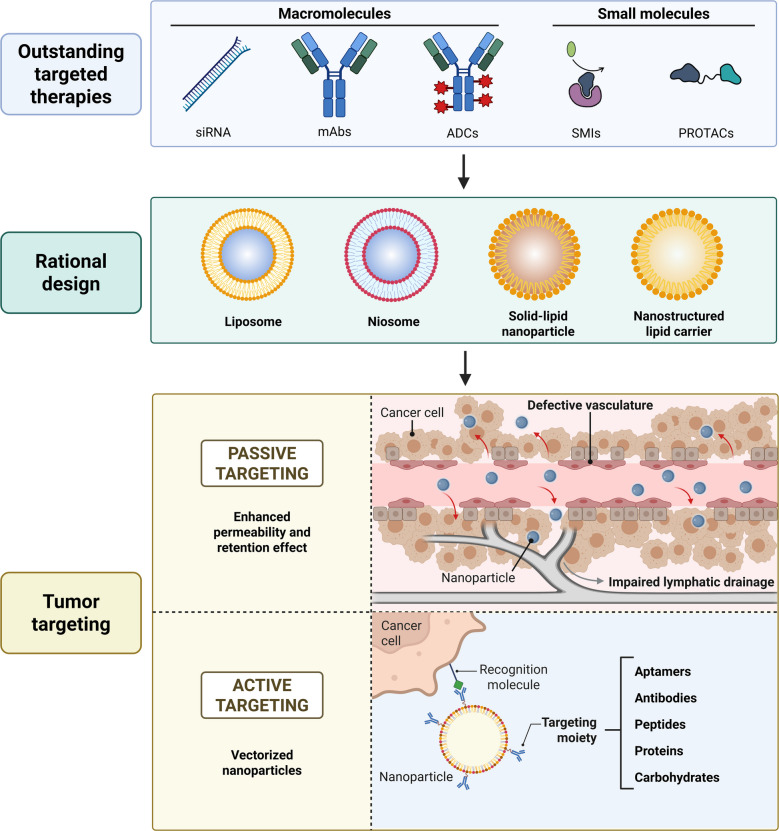

## Introduction

Cancer therapy has undergone a profound paradigm shift in recent years, evolving from conventional, non-specific treatments like chemotherapy to highly personalized, precise targeted therapies [1, 2]. Particularly within the last five years, the oncological therapeutic arsenal has expanded rapidly to address a broader array of targets. This evolution includes not only the consolidation of monoclonal antibodies (mAbs) and antibody–drug conjugates (ADCs), but also the accelerated clinical translation of small interfering RNAs (siRNAs), novel small-molecule inhibitors (SMIs), and the emergence of Proteolysis Targeting Chimeras (PROTACs) as revolutionary protein degraders [3].

However, recent translational studies demonstrate that the clinical performance of these advanced targeted therapies is frequently limited by a cascade of complex biological barriers rather than their intrinsic drug potency [1, 4]. For instance, delivering therapeutics through the dense tumor extracellular matrix and the basement membrane remains a significant challenge for large macromolecules [4]. Specifically, mAbs and ADCs often suffer from limited extravasation and heterogeneous penetration into solid tumors, as well as on‑target, off‑tumor toxicity due to widespread expression of their antigens in normal tissues. Conversely, nucleic acid-based drugs like siRNA face rapid clearance, enzymatic degradation, poor membrane permeability and frequent endosomal entrapment, whereas many SMIs and PROTACs are constrained by suboptimal solubility, low oral bioavailability and the emergence of resistance [4–6].

These pharmacokinetic (PK) and pharmacodynamic (PD) limitations underscore the need for delivery systems. Concurrently, the last few years have witnessed an unprecedented acceleration in the field of nanomedicine, accompanied by an explosion of research on drug delivery systems (DDS), largely driven by the global clinical validation of lipid-based nanoparticles (LBNPs) used in mRNA COVID-19 vaccines [6]. This historical milestone has strengthened LBNP research in oncology, establishing these nanocarriers as versatile platforms that can be tailored in terms of lipid composition, particle size, surface charge, stealth coating and active targeting ligands to address specific shortcomings of different classes of targeted anticancer agents.

Despite several reviews on cancer nanomedicine and on targeted therapies separately, there is a lack of integrative analyses specifically focused on how LBNPs can be rationally designed to overcome the different PK and PD limitations of these clinically relevant therapies. By integrating current knowledge on targeted therapies with emerging principles of LBNP engineering and recent progress in clinical translation, this review aims to provide a translationally oriented framework that links concrete clinical limitations with rational design guidelines for LBNPs.

## Targeted therapies: clinical context

**Fig. 1:**
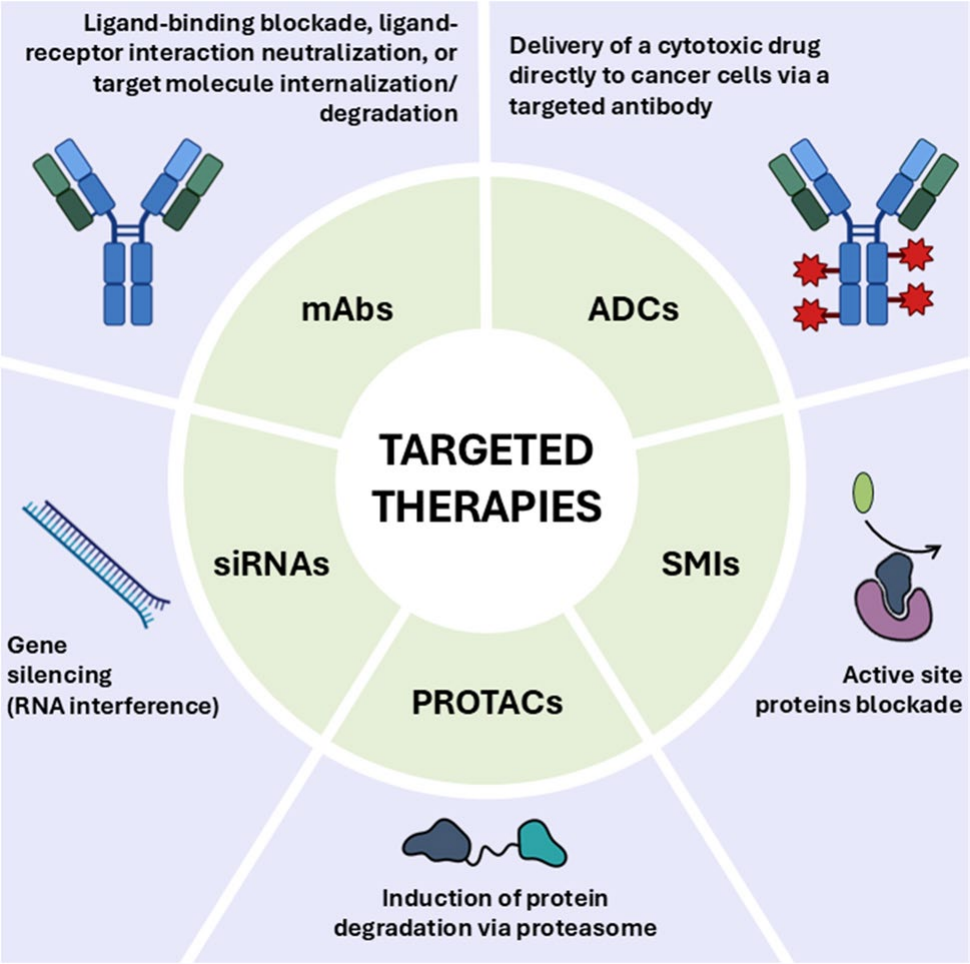
Overview of targeted therapies and their mechanisms of action. Abbreviations: monoclonal antibodies (mAbs), antibody–drug conjugates (ADCs), small interfering RNA (siRNA), small molecule inhibitors (SMIs) and PROteolysis TArgeting Chimeras (PROTACs)

Molecular targeted therapy refers to the use of drugs or other substances that block, inhibit, silence, and/or degrade specific oncogenic molecules (molecular targets) to block the growth, progression and metastasis of cancer cells. To date, targeted therapies encompass five main classes with distinct mechanisms: mAbs block extracellular ligands/receptors (ligand neutralization, receptor internalization); ADCs deliver cytotoxic payloads via antigen-specific internalization; siRNA silences genes through sequence-specific RNA interference; SMIs inhibit oncogenic kinases/proteins at catalytic sites; and PROTACs induce target protein ubiquitination/degradation via recruited E3 ligases (Fig. 1) [3].

Each modality offers unique clinical advantages but shares common PK limitations that LBNPs can address (Table 1). By 2026, > 60 mAbs are FDA/EMA-approved for cancer treatment, targeting HER2 + breast cancer (Trastuzumab, Herceptin®) and colorectal cancer (Cetuximab, Erbitux®), among others [7]. Despite high specificity, mAbs face high production costs, immunogenicity, poor tumor penetration and intravenous-only administration (Table 1) [1]. The FDA approved at least 15 ADCs, primarily targeting HER2 + cancers (Enhertu®, Kadcyla™) [7]. They combine antibody specificity with cytotoxic payloads but face low internalization efficiency, linker instability, and manufacturing challenges (Table 1) [1]. Patisiran (Onpattro®) was the first FDA-approved siRNA in 2018; and 7 more were approved by 2025, demonstrating remarkable gene-silencing specificity through RNA interference, all of them targeting hepatocytes [8]. Despite this promise, their clinical translation for oncology remains hindered by rapid nuclease-mediated degradation, poor cellular permeability, activation of innate immune responses, and endosomal entrapment (Table 1) [9]. SMIs, including Capivasertib for HR +/HER2- breast cancer and Encorafenib for BRAFV600E colorectal cancer, represent the most extensively approved category of drugs among all tumor treatment medication and offer clear advantages in oral bioavailability and cost over biologics [10–12]. Yet their clinical utility is constrained by poor aqueous solubility, rapid development of primary and acquired resistance, and on-target/off-tumor toxicity (Table 1) [10, 13, 14]. PROTACs such as ARV-471 (launched a Phase III clinical trial for patients with breast cancer) represent a paradigm shift by catalytically degrading 'undruggable' proteins via ubiquitin–proteasome recruitment [15]. However, their large molecular weight (> 1000 Da), poor membrane permeability, suboptimal solubility, and bell-shaped 'Hook effect' dose–response complicate clinical dosing. Broad target expression also raises on-target/off-tumor toxicity concerns (Table 1) [16–20].

**Table 1:** Summarized characteristics of main targeted therapies

Targeted therapy	Advantages	Disadvantages
mAbs	High specificity and affinity Reduced risk of side effects Fast response Long-term therapeutic activity	High production costs Immunogenicity Low penetration Intravenous administration Long half-life with risk of side effects
ADCs	High specificity Design flexibility Reduced immunogenicity	On-target, off-tumor toxicity Limited internalization Low efficiency Resistance Manufacturing difficulties
siRNA	High specificity Favorable safety profile High efficiency Versatility	Nuclease degradation Rapid clearance Immunogenicity Low permeability Endosomal entrapment Off-target effects
SMIs	PK profile Cost-effective Storage stability	Resistance “Undruggable” targets On-target, off-tumor toxicity Low bioavailability Short half-life
PROTACs	Recyclable Low dose efficacy High specificity Improved druggability Combat drug resistance Long half-life	Reduced bioavailability Limited permeability Hook effect On-target, off-tumor toxicity Possible proteostasis saturation

*mAbs* Monoclonal antibodies, *ADCs* Antibody–drug conjugates, *siRNA* Small interfering RNA, *SMIs* Small molecule inhibitors, *PROTACs* PROteolysis TArgeting Chimeras

These pharmacokinetic and pharmacodynamic limitations across all modalities highlight clear opportunities for LBNPs optimization, which will be systematically addressed in Table 3.

## Nanomedicine

Nanotechnology is defined as the science and engineering of manipulating matter at the nanoscale. This discipline has numerous applications, including fighting against diseases known as Nanomedicine. Using materials and devices at the nanoscale enables the development of new or improved diagnosis methods or treatments for different diseases. The ultimate goal is to enhance efficacy, reduce side effects and improve patient outcomes. In the field of drug delivery, the drug is encapsulated in nanoparticles (NPs) that function as DDS, effectively increasing the efficacy and reducing the toxicity of potentially outstanding drugs [21].

Over the past three decades, research in cancer nanomedicine has expanded significantly as DDS present several attractive properties in oncological applications. They enable targeted drug delivery at the cellular, organelle, and tissue levels. Consequently, the precise delivery of therapeutics to intracellular sites of action is facilitated, as well as side effects are reduced and the efficacy of drugs is increased, thereby improving the therapeutic index. DDS have the potential to improve the pharmaceutical properties of drugs, including tumor accumulation via the enhanced permeability and retention (EPR) effect, prolonging circulation time, improving solubility, and enhancing stability [22]. These systems can be designed for sustained release and stimulus-responsive drug release [23, 24]. Furthermore, nanomedicine allows for the co-delivery of a combination of drugs within a single carrier, which is particularly useful for overcoming multidrug resistance [25]. NPs’ adaptability extends to cancer diagnostics and the integration of diagnostic and therapeutic functions in a single platform, known as “theragnostics” [22]. Additionally, NPs offer innovative strategies for the development of synthetic vaccines and protect sensitive drugs against harsh environments, such as low pH and high protease activity [22].

Different materials have been explored over the years for the design of DDS. Lipid-based, polymer-based or metallic NPs have been designed for the targeted delivery of therapeutic nucleic acids, chemotherapeutic agents, and immunotherapies to tumors. Recent advances in nanotechnology, combined with a deeper understanding of cancer biology and nano-bio interactions, have driven the development of specialized nanocarriers that enhance therapeutic efficacy while reducing off-target toxicity through tumor tissue-, cell-, and organelle-specific targeting [21].

### Mechanisms of action of nanoparticles

The mechanism of cancer cells targeting can be achieved in two different ways: passively or actively. Furthermore, three generations of nanomedicines can be distinguished according to their targeting approaches [21]:

- *1*^*st*^ *generation*: tissue-specific targeting. Nanomedicines are designed to increase their accumulation within solid tumors and thus to reduce off-target effects. Primarily relies on passive targeting mechanisms, such as EPR effect.
- *2*^*nd*^ *generation*: cell-specific targeting. This approach focuses on achieving a selective and effective internalization of nanomedicines into tumor cells. It typically involves functionalizing NPs with targeting moieties, such as antibodies, antibody fragments, nucleic acid aptamers, peptides, carbohydrates, and small molecules, which can selectively bind to tumor-specific antigens or receptors overexpressed on the plasma membrane and promote cellular uptake of the conjugated NP. A novel biomimetic targeting strategy has gained significant interest, involving the coating of NPs with plasma membranes derived from cancer cells, blood cells, or stem cells. This approach enables homotypic or heterotypic adhesion, enhancing tumor cell recognition and interaction.
- *3*^*rd*^ *generation*: organelle-specific targeting. The aim is to precisely deliver the drug to specific organelles, such as the nucleus, mitochondria, or lysosomes, to maximize therapeutic efficacy while avoiding drug resistance.

#### Passive targeting

Tumor tissue targeting primarily relies on the EPR effect, which takes advantage of the leaky tumor vasculature and impaired lymphatic system. These physiological characteristics enable NPs to passively accumulate within solid tumors, enhancing drug delivery efficiency [21].

Compared to normal blood vessels, tumor vasculature exhibits several distinct abnormalities. These include: (1) broad angiogenesis; (2) a high proportion of proliferating endothelial cells, leading to structural deficiencies and irregular stromal basement membrane formation; (3) hypervasculature; (4) conflicting and violent bloodstream; and (5) slow venous return.

**Fig. 2:**
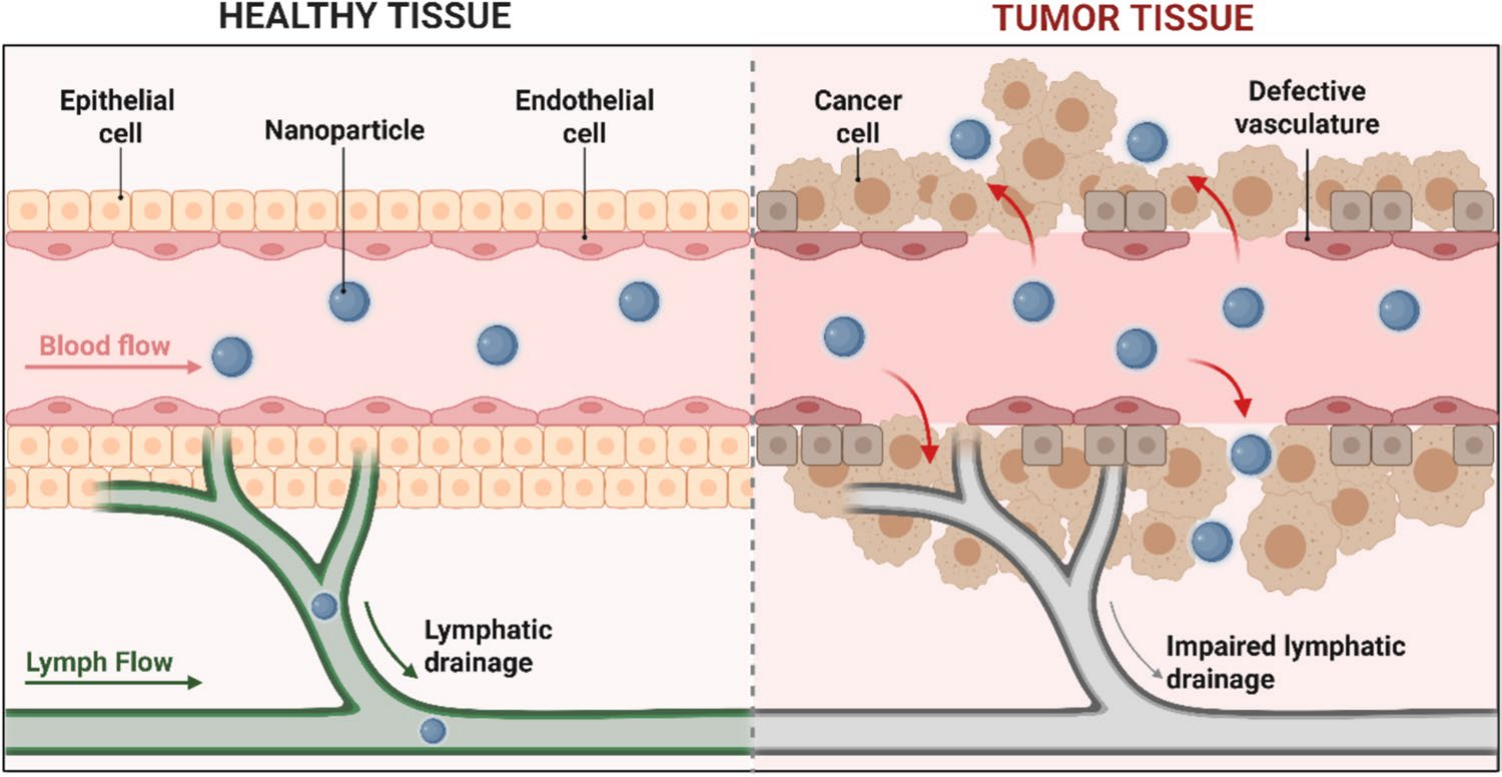
Passive targeting of nanoparticles to cancer cells

Most tumor capillaries display increased permeability and are often structurally compromised due to these anomalies. Since the blood vessels in many tumors have a leaky endothelium with gap sizes ranging from 400 to 600 nm, NPs between 10 and 500 nm can extravasate and accumulate within the tumor's interstitial space. This phenomenon represents the “enhanced permeability” component of the EPR effect. In addition to the increased vascular permeability, tumors also exhibit a dysfunctional lymphatic system, which contributes to inefficient waste drainage within tumor tissues. As a result, NPs accumulate inside the tumor since the impaired lymphatic system fails to effectively clear them. This phenomenon denotes the “enhanced retention” component of the EPR effect (Fig. 2) [21].

Although extravasation through endothelial gaps has traditionally been considered the main mechanism of NPs accumulation, recent studies suggest additional pathways, such as transcellular transport and via endocytosis/transcytosis. Delivering NPs systemically poses challenges due to the rapid and extensive blood circulation, requiring NPs to persist in circulation long enough to reach tumors. Even after extravasation, NP penetration into the tumor core can take hours or days, emphasizing the need for an optimized design. Key physicochemical properties such as size, shape, mechanical properties, surface chemistry, and surface charge affect NP interactions with cells and proteins, influencing both efficacy and toxicity [22].

Of note, all clinically approved nanomedicines rely on EPR-mediated passive tumor targeting (Table 2). In this sense, anti-tumor NPs have been successfully used in clinical practice, taking advantage of the EPR effect to improve the PK profile of encapsulated drugs. Due to their leading role in clinical application and a greater number of regulatory approvals, LBNPs represent the most successful formulations based on the EPR effect. Among approved LBNPs, PEGylated liposomal Doxorubicin (Doxil®/Caelyx®) stands as the pioneering example of exploiting the EPR effect to improve the PK profile. A comprehensive list and detailed discussion of these clinically approved EPR-dependent LBNPs is provided in section "Clinically approved lipid-based nanomedicines" and Table 2. Collectively, these clinically approved nanomedicines demonstrate that exploiting the EPR effect can effectively improve pharmacokinetics, reduce systemic toxicity and, in specific indications, enhance antitumor efficacy compared with conventional formulations.

**Table 2:** Approved drug delivery systems (DDS) in clinical use for treating cancer [26–29]

			Date of approval		
DDS	Therapeutic agent	Brand name	FDA	EMA	Clinical use
Lipid-based NPs	Doxorubicin	Doxil®	1995	-	Ovarian cancer and breast cancer
		Caelyx®	-	1996	Ovarian cancer and breast cancer
		Myocet®	-	2000	Breast cancer
	Daunorubicin	DaunoXome®	1996	-	Kaposi’s Sarcoma
	Daunorubicin and cytarabine	Vyxeos®	2017	2018	Myeloid leukemia
	Mifamurtide	Mepact®	-	2009	Osteosarcoma
	Vincristine	Marqibo®	2012	-	Acute lymphoblastic leukemia
	Cytarabine	DepoCyt®	1999	2001 (withdrawn in 2018)	Lymphomatous meningitis
	Irinotecan	Onivyde®	2015	2016	Pancreatic cancer
	Paclitaxel	Apealea®	-	2018 (withdrawn in 2024)	Ovarian cancer
Polymer-based DDS	L-asparaginase	Oncaspar®	1994	2016	Acute lymphocytic leukemia
	Leuprolide/Leuprorelin	Camcevi®	2021	2022	Prostatic cancer
		Eligard®	2002	2007	Prostatic cancer
	Goserelin	Zoladex®	1989	1987*	Prostatic cancer
Metallic NPs	NBTXR3 (hafnium oxide)	Hensify®	-	2019	Squamous cell carcinoma
	Iron oxide	NanoTherm®	-	2010	Glioblastoma multiforme
Protein-based NPs	Paclitaxel	Abraxane®	2005	2008	Pancreatic cancer, breast cancer and NSCLC
		Pazenir®	-	2019	Pancreatic cancer, breast cancer and NSCLC
	Sirolimus	Fyarro®	2021	-	Sarcoma
	Denileukin diftitox	Ontak®	1999	-	T-cell lymphoma

*FDA* American Food and Drug Administration, *EMA* European Medicines Agency, *NSCLC* Non-small cell lung cancer

*Zoladex was nationally authorized in United Kingdom before EMA establishment

The EPR effect was first described in 1986 by Matsumura and Maeda [30]. They demonstrated that macromolecules of a certain molecular range preferentially accumulate in solid tumors via this unique phenomenon, which has paved the way for tumor tissue-targeted delivery of macromolecular drugs and nanomedicines. Nevertheless, it is now widely agreed that the EPR effect is far more complex than initially described. Its variability is significant, not only between patients but also across different tumor types, due to inherent differences in tumor genetics, the tumor microenvironment, and the physicochemical properties of NPs [21]. Consequently, the clinical performance of EPR-dependent nanomedicines is highly context-dependent and does not translate into uniform benefit across all tumor types and patients.

Despite its central role in the development of first-generation nanomedicines, the EPR effect is now recognized as a highly heterogeneous and often inefficient mechanism in human tumors, influenced by both inter- and intra-tumoral conditions [21]. Recent reviews have emphasized that the clinical performance of EPR-based nanomedicines remains inconsistent despite decades of encouraging preclinical data, as clinical and translational studies indicate that only a small fraction of the injected NP dose actually accumulates in solid tumors (typically less than 1%), and this accumulation varies markedly between tumor types and individual patients, reflecting differences in vascular density, stromal composition and interstitial pressure [31–33]. Fibrotic, poorly perfused malignancies such as pancreatic adenocarcinoma typically display minimal EPR-mediated uptake, whereas more vascularized tumors show more favorable but still heterogeneous accumulation. Moreover, patient-specific factors—including tumor size, anatomical location, immune activation and inflammatory status—further modulate NP delivery and clearance, explaining the gap between highly permeable preclinical models and human tumors [31].

As a result, relying exclusively on passive, EPR-driven targeting as a universal delivery paradigm provides limited and unpredictable benefit across diverse tumor types and patients, rather than explaining all observed clinical successes with nanomedicines [34]. These limitations have motivated the development of alternative strategies [34–37]:

- *Receptor-mediated transcytosis*, an active transport mechanism that exploits ligand-receptor interactions at the tumor endothelium to drive NP crossing of the vascular barrier and improve intratumoral penetration, particularly in stroma-rich tumors with limited EPR [38, 39].
- *Pharmacological or physical priming*—such as angiogenic factors, erythropoietin to enhance tumor perfusion, corticosteroids for extracellular matrix remodeling, and vasodilators or blood pressure modulation to improve microvascular flow—aim to overcome EPR heterogeneity and enhance nanomedicine delivery [40, 41].
- *Immune-modulating combinations* to remodel the tumor immune microenvironment, reprogram immunosuppressive cells, and synergize with checkpoint inhibitors or other immunotherapies to convert immunologically 'cold' tumors into responsive lesions [42, 43].
- *Active targeting*, which harness ligand-receptor interactions to augment the accumulation of nanodevices at action sites, promoting enhanced retention, cellular internalization and penetration beyond passive EPR accumulation alone [44].

#### Active targeting

**Fig. 3:**
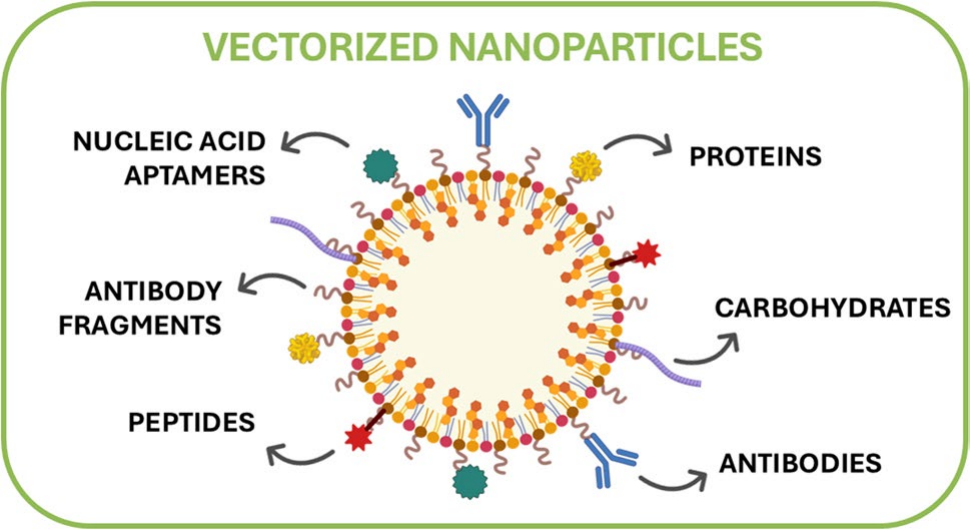
Active targeting of nanoparticles using conjugating ligands

In addition to passive targeting, active targeting techniques represent an alternative to enhance the accumulation of nanomedicines in low-EPR tumors and to be more selective to cancer cells. In active targeting, specific ligands that are detected by disease-site cells are attached to the exterior of NPs, enabling them to interact with tumor cells directly [21]. Conjugating ligands is the most outstanding tumor cell-specific targeting technique. The different moieties employed in this technique include antibodies, antibody fragments, aptamers, proteins, peptides or carbohydrates (Fig. 3) [21].

NPs can be linked to a ***mAb***, ensuring precise drug delivery targeting different tumor-specific antigens or proteins. Typical targets of this antibody conjugated NPs (ACNPs) are EGFR, HER2 and prostate-specific membrane antigen (PSMA). EGFR is overexpressed in several solid tumors, including NSCLC, breast cancer, ovarian cancer, and head and neck squamous cell carcinoma (HNSCC). EGFR mAb-decorated NPs showed superior tumor-targeting capacity and enhanced antitumor efficacy in preclinical models and clinical trials [21]. For instance, the functionalization of NPs with cetuximab or trastuzumab has successfully enhanced tumor accumulation and cytotoxicity in HNSCC and breast cancer models [45, 46]. The translational potential is further evidenced by clinical trials, such as the TargomiRs minicells, where EGFR-targeted delivery of miRNA achieved clinical response in Phase I trial for malignant pleural mesothelioma [47].

**Fig. 4:**
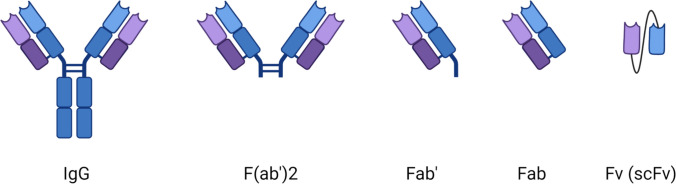
Different components of an antibody

***Antibody fragments*** were proposed as targeting moieties for selective NPs delivery. These molecules retain at least one antigen-binding region from mAbs while circumventing their associated challenges. An antibody can be cleaved into several different fragments, including the antigen-binding fragment (Fab), Fab’, and F(ab’)_2_. With the use of genetic engineering and phage display techniques, a variety of engineered antibody fragments, such as the single-chain variable fragment (scFv) or the single domain antibody (sdAb), can be obtained (Fig. 4). Antibody fragment-functionalized NPs have demonstrated their potential, and some promising candidates, including C225-ILs-DOX (anti-EGFR Fab’), MM-302 (anti-HER2 scFv), and SGT-53 and SGT-94 (anti-TfR scFv), have entered clinical trials [21]. Furthermore, a Phase II clinical trial (NCT00470613) is evaluating a p53 plasmid encapsulated into liposomes conjugated to SGT-53 for treating pancreatic cancer [48].

***Nucleic acid aptamers***, often referred to as "chemical antibodies”, are short, single-stranded DNAs or RNAs that are generated for binding to a wide range of targets, from small molecules to whole cells, with high specificity and affinity. Representative targets of these molecules are the cell membrane-located biomarkers PSMA, epithelial cellular adhesion molecule (EpCAM) and mucin-1 (MUC1) [21]. Several preclinical studies have demonstrated that aptamers have impressive potential in active targeting against these biomarkers, showing improved antitumor efficacy and specific targeting in in vitro and in vivo cancer models, such as prostate or breast cancer [49–51].

Many ***non-antibody proteins*** can bind cell-surface molecules with high affinity and specificity, and therefore, have the potential to be utilized for precise transportation of NPs to cancerous cells. For instance, transferrin is the most extensively studied targeting protein and has been used to specifically target the plasma membrane-located transferrin receptor, which shows increased expression in various types of cancer [21]. This has been demonstrated in preclinical strategies for glioma therapy and further validated in clinical trials (NCT00689065), where targeted NPs achieved specific tumor accumulation for siRNA-based treatments in solid tumors [52, 53].

***Peptides*** are linear, branched, or cyclic amino acid chains that are typically limited to 50 residues or less. Their smaller size offers several advantages such as ease of synthesis and conjugation, improved stability and biocompatibility, as well as increased surface loading, resulting in enhanced targeting efficiency. The most investigated peptide ligands are Arg-Gly-Asp (RGD)-based peptides, capable of specifically and strongly binding to the receptor integrin α_v_β_3_, which is overexpressed in tumor cells and their supporting vasculature [21]. Conjugating NPs with RGD peptides has proven effective in inhibiting glioblastoma cell growth by specifically targeting integrin receptors [54].

Owing to the excellent biocompatibility and specific recognition of carbohydrates by certain cell-surface proteins, ***carbohydrate moieties*** have gained significant popularity as targeting ligands for precisely delivering NPs to tumor cells in recent decades. Some relevant examples of this vectorizing carbohydrates include galactose, β-galactoside, mannose and hyaluronic acid (HA), which target the asialoglycoprotein receptor, galectins, the mannose receptor, and CD44, respectively. HA can specifically bind to two major receptors, CD44 and RHAMM, which are overexpressed on the surface of various tumor cells compared with normal cells [21]. HA-decorated nanomicelles exhibited a significant targeting effect towards CD44, resulting in substantial tumor growth inhibition and improved therapeutic efficacy for treating breast cancer [55].

In addition to the aforementioned conjugating ligands, the employment of several small molecules, including folic acid, biotin, and glycyrrhetinic acid, has been explored for the functionalization of NPs [21].

#### Stealth coating

Moreover, although targeting ligands are crucial for the accurate delivery of nanocarriers into tumor cells, nonspecific interaction of vectorized or non-vectorized NPs with components from biological environments or undesired recognition by immune cells during circulation will greatly compromise their efficiency.

Upon entering the bloodstream, NPs can adsorb plasma proteins and form “protein corona”. This corona is classified into soft corona, which consists of proteins with lower affinity and higher tendency to desorb from NPs’ surfaces, and the hard corona, a more stable layer of tightly bound proteins that plays a crucial role in determining the behavior and in vivo fate of NPs. The presence of a protein corona can make NPs vulnerable to the immune system's recognition and clearance. Furthermore, active targeting ligands may also be buried by the protein corona, resulting in their ineffectiveness and leading to off-target effects [56, 57].

The most common approach to limiting immune system clearance and minimizing the formation of the protein corona is PEGylation of the nanoparticle surface, a process known as “stealthing”. Poly(ethylene glycol) (PEG) is considered the gold standard for stealth polymers. PEGylated nanomedicines are well-known for their ability to prolong blood circulation and efficiently accumulate at tumor sites via the EPR effect. However, this approach can also compromise the interaction between nanomedicines and cell membranes, resulting in poor cellular internalization and unsatisfactory tumor inhibition, a phenomenon commonly referred to as the “PEG dilemma”. The effectiveness of PEG functionalization depends on both the length of the polymer chains and their density at the surface [56]. In clinical applications, it has been observed that PEGylated particles can trigger the production of anti-PEG antibodies, further accelerating clearance. This underscores the need for alternative strategies to reduce in vivo NPs clearance. Promising alternatives include surface coatings with zwitterionic species and carbohydrates, as part of a design framework aimed at maintaining a favorable balance between circulation, immune recognition, targeting performance and regulatory acceptability [22, 56].

Despite the enhanced therapeutic properties of vectorized nanomedicines, the translation of ligand-decorated NPs into approved anticancer therapies remains limited. In vivo, the formation of a dynamic protein corona can mask targeting ligands and alter NPs identity, while the heterogeneous receptors in tumors, together with the additional complexity introduced by ligand conjugation, poses significant challenges for patient stratification, large-scale manufacturing and regulatory approval. Moreover, once NPs reach the tumor, the majority are internalized via endocytic pathways, which frequently results in endosomal entrapment; thus, rational design of actively targeted NPs must concurrently address protein corona effects, biologically meaningful target selection and efficient endosomal escape to achieve consistent clinical benefit.

#### Endosomal escape

Extracellular macromolecules and particles may be internalized into cells via endocytosis. The endocytic pathway is divided into two different mechanisms: phagocytosis and pinocytosis. The uptake through endocytosis in both mechanisms involves the internalization of the substance into an endocytic vesicle formed through plasma membrane fold, fusion into the early endosomal compartment, maturation into a late endosome, and accumulation in the lysosome. It is known that intracellular internalization of most therapeutic compounds, including NPs, occurs via endocytosis [58].

**Fig. 5:**
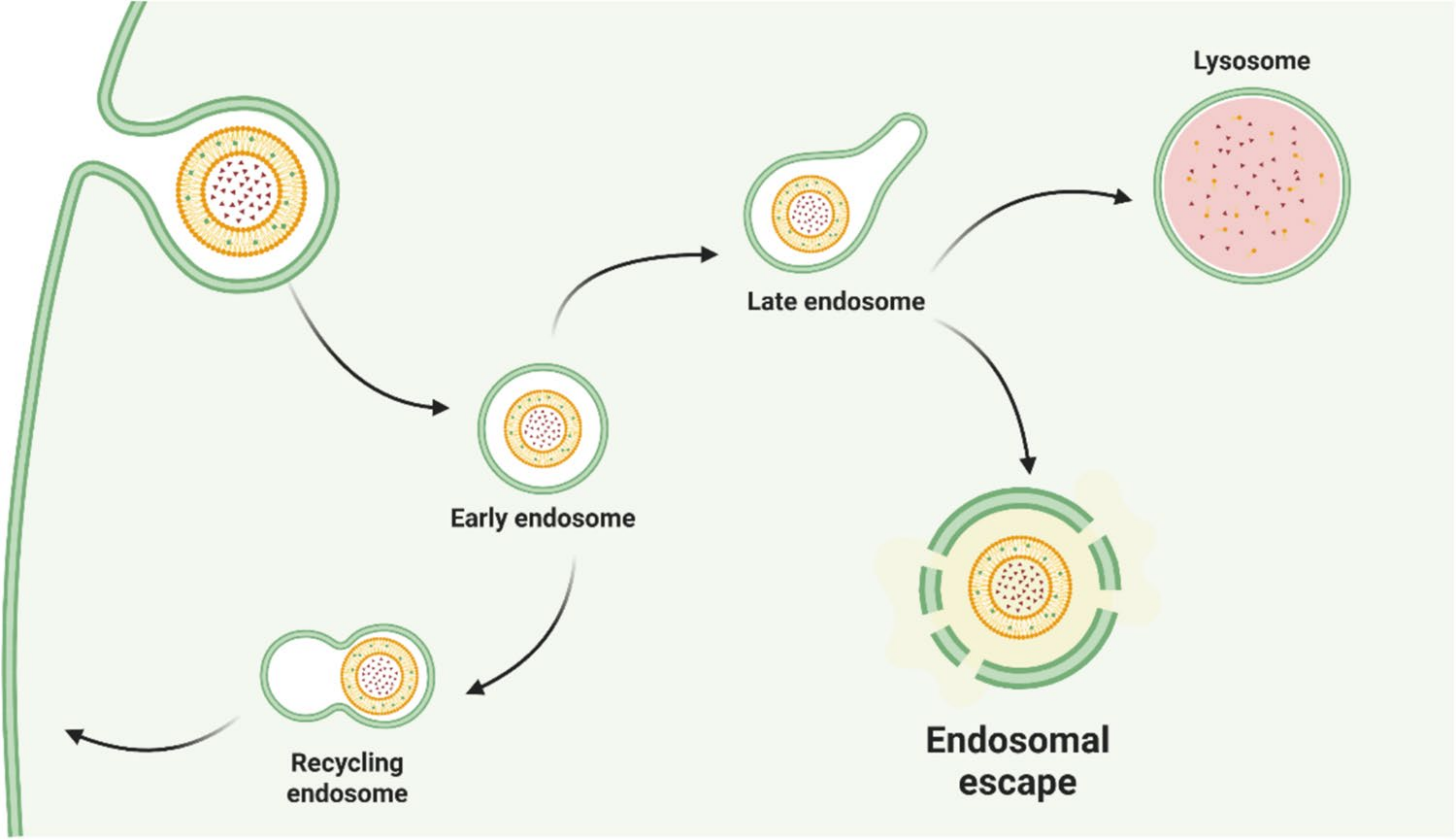
Schematic diagram of endosomal escape

During maturation of the endosome, the pH decreases from the physiological pH of 7.4, down to ∼pH 6.5 in the early endosome, ∼pH 6.0 in the late endosome, and ∼pH 5.0 in the lysosome, where the majority of the foreign materials are degraded enzymatically (Fig. 5). For efficient and improved delivery, the escape from endosomes before lysosomal maturation is a crucial stage. Endosomal escape is considered a bottleneck in the field of nanotechnology. Therefore, it is important to understand the process to develop more effective drug delivery systems. Membrane fusion, membrane destabilization, particle swelling, and osmotic rupture, have been hypothesized as mechanisms of escaping the endo/lysosomal pathway. However, at present, there is no consensus on the mechanism of endosomal escape processes, and there is no capability to predict a nanoparticle's formulation that will enable efficient endosomal escape [59]. Future translational development of LBNPs for targeted therapies delivery will increasingly rely on the integration of quantitative mechanistic tools to assess endosomal escape and systematic structure–activity relationships for ionizable lipids and helper components.

### Building materials: options for clinical translation

**Fig. 6:**
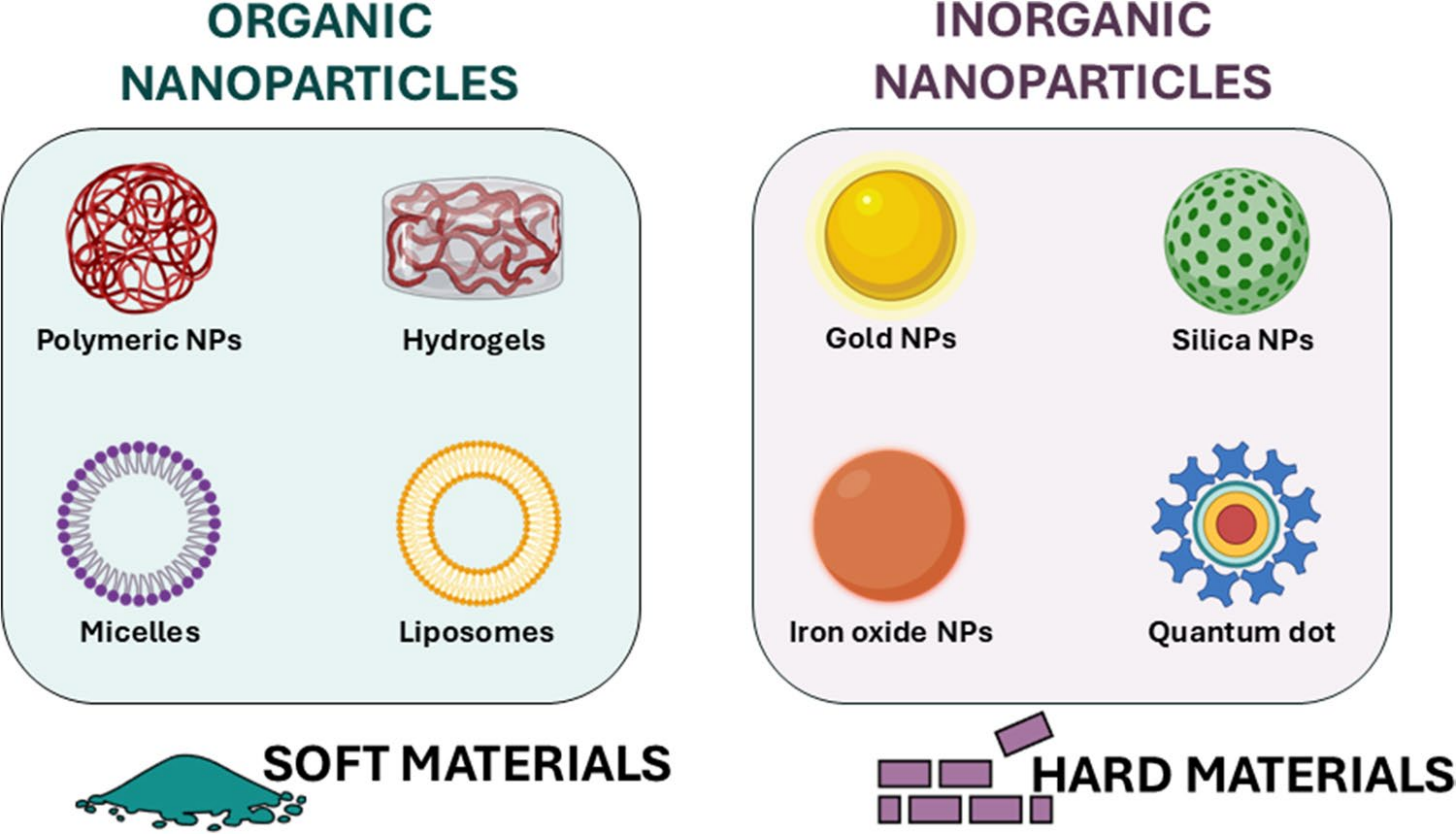
Classification of nanoparticles (NPs) based on building materials

DDS vary in size, composition, and functionality. However, they are generally composed of a central matrix, a therapeutic payload, and, in many cases, surface modifications. Currently, nanotechnology-based DDS can be formulated from soft (organic NPs: polymeric, liposomes, micelles, etc.) to hard materials (inorganic NPs: gold, silica, iron oxide, etc.) (Fig. 6).

To date, several DDS have already been approved for clinical use (Table 2), and many others have demonstrated significant potential in preclinical studies or are under clinical trials. The success of any new cancer nanomedicine depends primarily on its safety and efficacy. Enhancing the safety profile and maximizing NPs accumulation at the tumor site are critical factors for clinical translation. However, achieving sufficient accumulation remains a major challenge due to the biological barriers that hinder NPs transport to the disease site [22].

The majority of clinically approved nanomedicines for cancer therapy via intravenous administration are LBNPs. However, other DDS, such as polymer-based and metallic NPs, have also successfully entered the clinic [26, 27]. Among innovative delivery systems, polymeric hydrogels also stand out due to their high-water content, elasticity, and three-dimensional porous network, making them highly attractive for drug delivery applications [60]. The current focus in the field is on passively targeting ability, based on the EPR effect, with a few clinical trials investigating ligand-mediated active [61, 62]. Unfortunately, clinical results showed only limited improvement with the ligand-mediated targeting strategies employed to enhance efficacy. This may be due to the adsorption of protein corona, which shields the ligands on NPs [28].

#### Hard materials-based NPs

Inorganic materials, such as metals and silica, are widely used to synthesize nanostructured materials with unique physical, electrical, magnetic, and optical properties. The precise formulation of these NPs is crucial for achieving the desired size, structure, and geometric shape, including nanospheres, nanorods, nanoshells, and nanoboxes [26].

Iron oxide NPs are the most commonly FDA-approved inorganic NPs, primarily for iron replacement therapy. Outstanding examples include Ferinject® (colloidal Fe(OH)_3_ NPs stabilized by carboxymaltose, used for anemia treatment) and Feraheme® (polymer-coated superparamagnetic iron oxide NPs formulated with mannitol, indicated for iron deficiency anemia in chronic kidney disease) [26, 27]. In the field of cancer treatment, the European Medicines Agency (EMA) approved in 2010 the use of NanoTherm® (iron oxide NPs) for treating glioblastoma multiforme, and the use of Hensify® (hafnium oxide NPs) for squamous cell carcinoma treatment in 2019 (Table 2) [29].

While inorganic NPs show promise in diagnostics and cancer therapy (mainly focused on hyperthermia treatments), their use in drug delivery remains limited due to biodegradability issues and rapid clearance. Future improvements may help overcome these barriers for metal-based NPs development [26].

#### Soft materials-based NPs

Polymeric NPs can be synthesized using natural or synthetic polymers. While natural biodegradable polymers like alginate and chitosan offer biocompatibility, their high immunogenicity and batch variability limit their clinical use. In contrast, synthetic polyesters, such as FDA-approved poly(lactic-co-glycolic acid) (PLGA), provide better control over drug loading, release kinetics, and stability. Various synthesis methods, including emulsification, nanoprecipitation, ion gelation, and microfluidics, yield NPs with distinct properties. Polymeric NPs can form nanospheres, where drugs are retained in the polymer matrix, or nanocapsules, where drugs are enclosed in a shell. These systems enable the delivery of hydrophilic and hydrophobic compounds, including small molecules, proteins, and vaccines [26].

Genexol-PM®, a paclitaxel-loaded nanoparticle formulation using PEG and poly-D,L-lactide (PDLLA), is approved for the treatment of breast cancer, NSCLC, and ovarian cancer in several Asian countries. However, it has not received approval from the FDA or EMA. As of now, there are no FDA or EMA-approved cancer treatments based on polymeric NPs [27]. Conversely, Oncaspar® or Eligard® constitute notable examples of polymer-based DDS approved by EMA and FDA for acute lymphoblastic leukemia and prostate cancer treatment, respectively (Table 2).

On the other hand, LBNPs are highly biocompatible, biodegradable, and effective DDS. They are typically spherical structures composed of a lipid bilayer enclosing an aqueous core, making them suitable for transporting both hydrophilic and hydrophobic drugs. Their advantages include easy formulation, high bioavailability, controlled drug release, and tunable biological, physical, and chemical properties [6]. Since 90% of developing pharmaceuticals and 40% of approved drugs are hydrophobic, there is significant interest in LBNPs for their delivery (Table 2). LBNPs have been widely used to enhance the bioavailability and controlled release of hydrophobic drugs, proving their potential as next-generation drug delivery systems. Encapsulating hydrophilic drugs remains challenging due to their high-water solubility and potential leakage. However, optimized formulation methods can achieve high encapsulation efficiency [6, 63].

## Lipid-based nanoparticles

### Classification and advantages

**Fig. 7:**
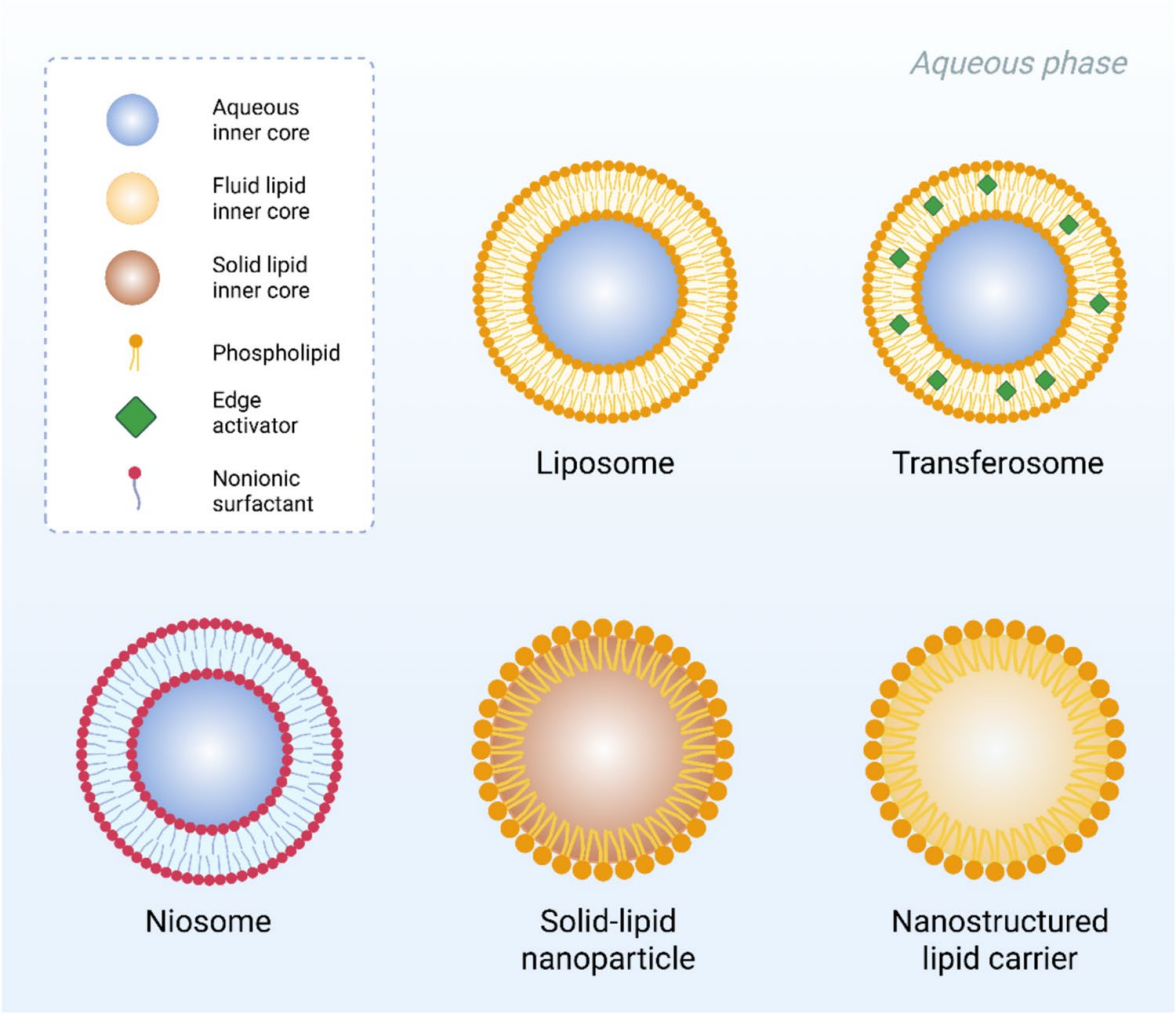
Schematic illustration of lipid-based nanoparticles

LBNPs are classified into different categories based on their nanostructure (Fig. 7): liposomes, transferosomes, niosomes, solid-lipid nanoparticles (SLNs) and nanostructured lipid carriers (NLCs). LBNPs present a wide range of advantages inherent to their composition and structure. Physicochemical properties of lipids make LBNPs an ideal DDS to improve the aqueous solubility, bioavailability, and therapeutic efficacy of drugs [64].

**Fig. 8:**
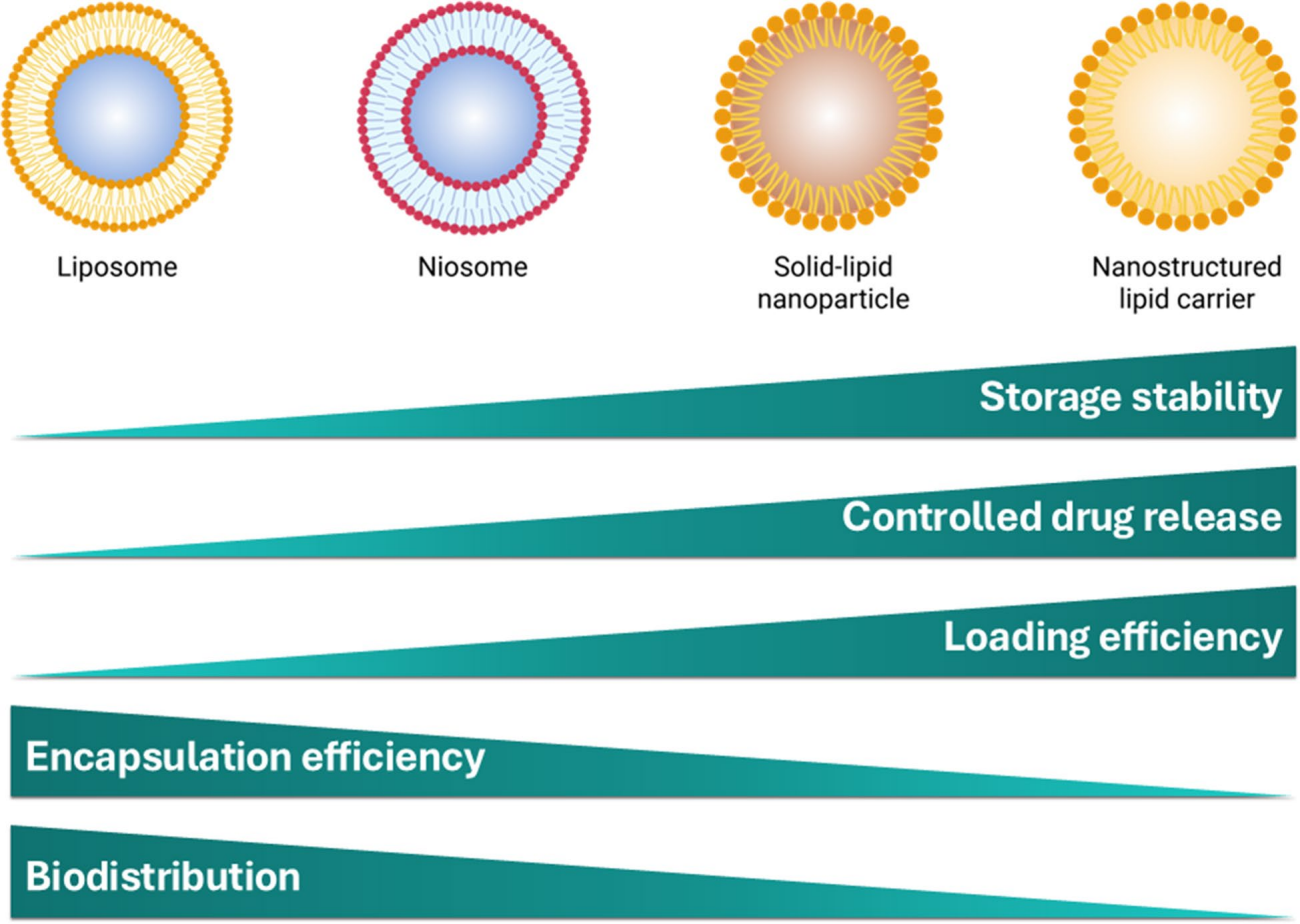
Differential characteristics of main lipid-based nanoparticles

***Liposomes*** are considered one of the most important DDS for their biocompatibility and biodegradability, and represent the first generation of LBNPs [65]. Liposomes are vesicular structures composed of one (unilamellar) or multiple (multilamellar) phospholipid bilayers with amphipathic properties that enclose a central aqueous compartment. They are often stabilized with cholesterol and other additives to enhance membrane rigidity and hydrophobic drug permeability [6]. Liposomes can incorporate hydrophobic and hydrophilic drug molecules, in the lipid bilayer or in the aqueous core, respectively (Fig. 8). A liposomal formulation differs from a nanoemulsion, which is an oil-in-water liquid dispersion or water-in-oil with one or more surfactants [65]. Ultra-deformable liposomes are called ***transferosomes***, which are composed of phospholipids and edge activators (such as surfactants) typically used in transdermal administration [66].

***Niosomes***, similarly to liposomes, are bilayer vesicles formed by nonionic surfactants. Despite their main composition is based on nonionic surfactants, they are classified as LBNPs because of the structure similarity to liposomes and the common addition of lipids such as cholesterol for their formulation [67]. Niosomes have greater stability than liposomes over a long period because of nonionic surfactants, which make them more stable with prolonged durability. In addition, compounds with solubility issues have improved dispersion, enhanced penetration when transdermal administration and a sustained release. Niosomes have other advantages like non-toxicity, easy and inexpensive fabrication method, simple storage and feasible large-scale production (Fig. 8) [64, 67].

***SLNs*** are nanosized colloidal DDS composed of a crystalline matrix made of complex glycerides and fatty acids, which are stabilized by emulsifiers and where the drug is entrapped. These NPs remain solid at both room and body temperature because of the solid-lipid composition [65]. Depending on the drug incorporation model, SLNs are classified into three different types: (1) solid solution model, when the drug is dispersed uniformly in the homogeneous lipid matrix; (2) drug-enriched shell model, when the drug is concentrated in the outer shell of the lipid NPs after recrystallization; and (3) drug-enriched core model, when the drug is entrapped in the inner core during crystallization [64]. In addition to their biocompatibility, SLNs are capable of loading both hydrophobic and hydrophilic drugs with slow water absorption. Also, they present higher stability and loading capacity of hydrophobic drugs over liposomes (Fig. 8) [68].

***NLCs*** are second-generation SLNs which include a mixture of liquid and solid lipids in different proportions that remain solid at body temperature. The addition of a liquid oil disrupts the crystalline matrix and reduces the melting point of the lipidic core, thus increasing the drug load capacity (drug expulsion during crystallization process is decreased) and storage stability of the system (Fig. 8) [65].

### Composition of lipid-based nanoparticles

LBNPs are composed of different components, depending on the specific type. The most commonly used components are as follows.

***Lipids*** have structurally diverse architectures characterized by a hydrophilic headgroup and one or more hydrophobic hydrocarbon tails. The headgroup can be negatively or positively charged, or zwitterionic, which influences LBNPs stability by promoting electrostatic repulsion between vesicles. The hydrophobic tails vary in chain length, symmetry, and degree of saturation, while the lipid backbone typically consists of phosphate, glycerol, or sphingosine. Together, these chemical characteristics regulate LBNPs properties such as bilayer formation, pH responsiveness, stability, and drug encapsulation and release; especially for SLNs and NLCs [69]. Commonly used lipids are medium chain triglycerides like Miglyol® or Softisan®, glycerides like Precirol® and fatty acids like stearic acid, palmitic acid or oleic acid [68, 70].

Natural ***phospholipids*** such as phosphatidylcholine and phosphatidylethanolamine are major components of mammalian cell membranes. Phospholipids with long, saturated acyl chains (like soy or egg lecithins) confer greater in vivo stability than those with short, unsaturated chains. Due to their endogenous origin, natural lipids are highly biocompatible and suitable for therapeutic applications. Alternatively, synthetic phospholipids such as 1,2-dioleoyl-sn-glycero-3-phosphocholine (DOPC), 1,2-dioleoyl-sn-glycero-3-phosphoethanolamine (DOPE), and dioleoyl phosphatidylglycerol (DOPG) are widely used for their purity, reproducibility, and structural similarity to natural ones, offering a reliable option for LBNPs formulation [69].

***Sterols*** are a class of natural lipid molecules found in most living organisms and are classified into phytosterols, zoosterols, and mycosterols, depending on whether they originate from plants, animals, or microorganisms. Cholesterol, an endogenous amphiphilic zoosterol, is a critical structural component of mammalian cell membranes and contributes to membrane integrity and lipid rafts functionality. In LBNPs formulations, the inclusion of cholesterol, typically in the range of 20–50 mol%, has shown enhanced in vivo stability and reduced bilayer permeability, thereby supporting prolonged and controlled cargo release. However, higher cholesterol content may also reduce encapsulation efficiency [69].

***Surfactants***, defined as molecules that reduce surface tension, are widely used in LBNPs formulations to enhance membrane flexibility, permeability, stability and drug delivery efficiency. Sodium cholate, Tween 60/80, and Span 60/80 are surfactants classified as edge activators. They destabilize the lipid bilayer, enhancing dermal and transdermal penetration, while their charge can also be exploited for electrostatic interactions with therapeutic molecules [69]. Non-ionic surfactants, which are amphiphilic and uncharged, provides stability, low toxicity, and ability to improve solubility and control drug release [67]. Beyond their classical surface activity, certain non-ionic surfactants, such as poloxamers like Pluronic® and poloxamines like Tetronic®, also function as hydrophilic surface modifiers. These block PEG-poly(propylene oxide) (PPO) copolymers are employed to reduce opsonization through surface “stealthing” [65]. Gemini surfactants, characterized by their dimeric structure with twin hydrophilic heads and hydrophobic tails connected by a flexible spacer, have emerged as next-generation carriers in cancer therapy, offering superior physicochemical properties such as low critical micelle concentration, enhanced nucleic acid complexation and reduced immunogenicity compared to conventional single-chain surfactants, while enabling highly efficient self-assembly and stabilization of LBNPs for targeted drug and gene delivery [71].

### Methods of formulation

**Fig. 9:**
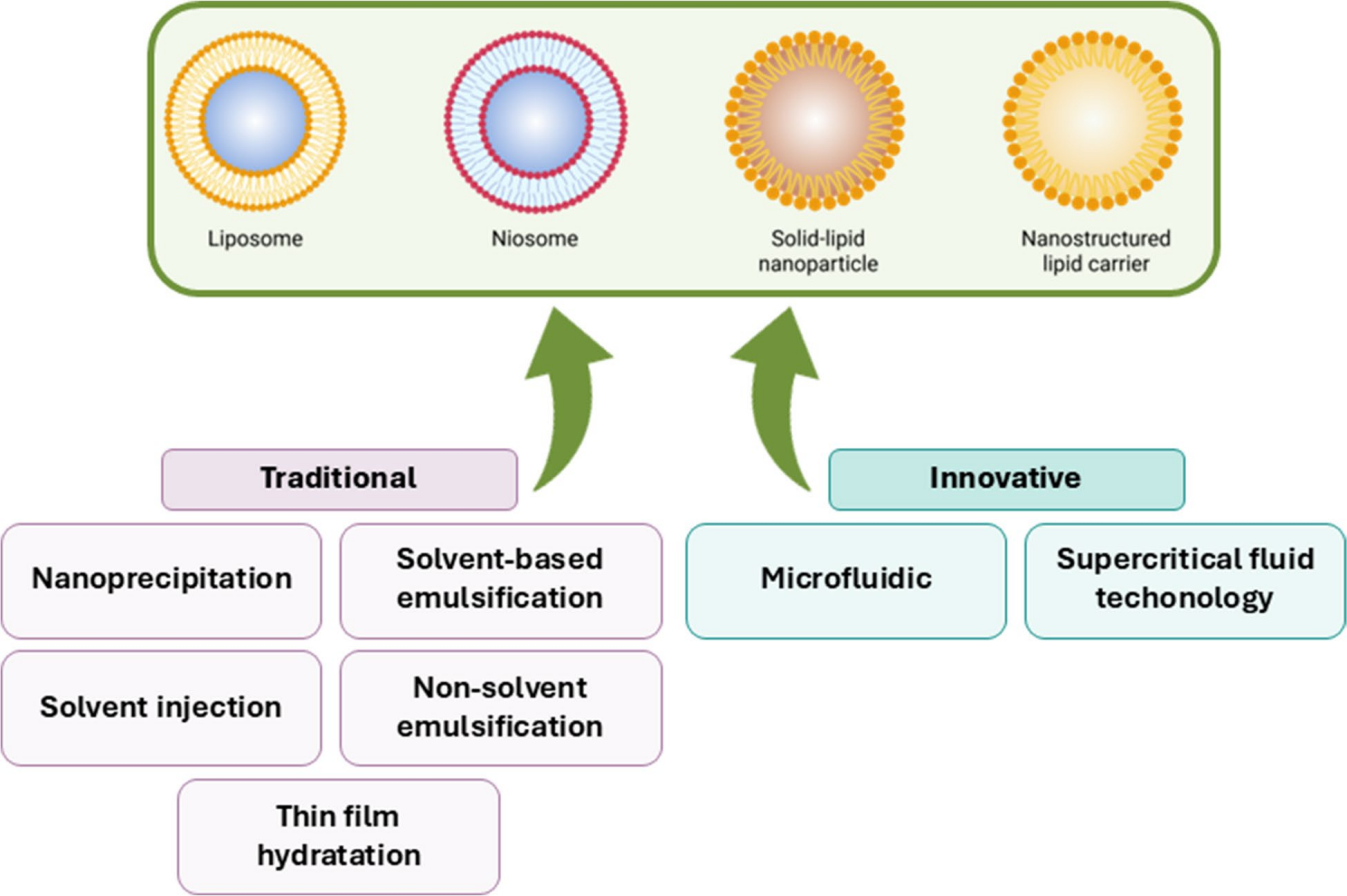
Classification of main formulation methods for lipid-based nanoparticles

LBNPs can be formulated using a wide range of formulation methods that can be classified in conventional or traditional methods (nanoprecipitation, solvent injection, solvent-based emulsification, non-solvent emulsification or thin film hydration) and innovative methods (microfluidic or supercritical fluid technology) (Fig. 9). Although simple to implement, conventional methods have significant limitations and typically require additional downstream processing, such as product homogenization. As a result, alternative and more advanced production techniques have been explored over time to meet industrial needs [72].

#### Nanoprecipitation

**Fig. 10:**
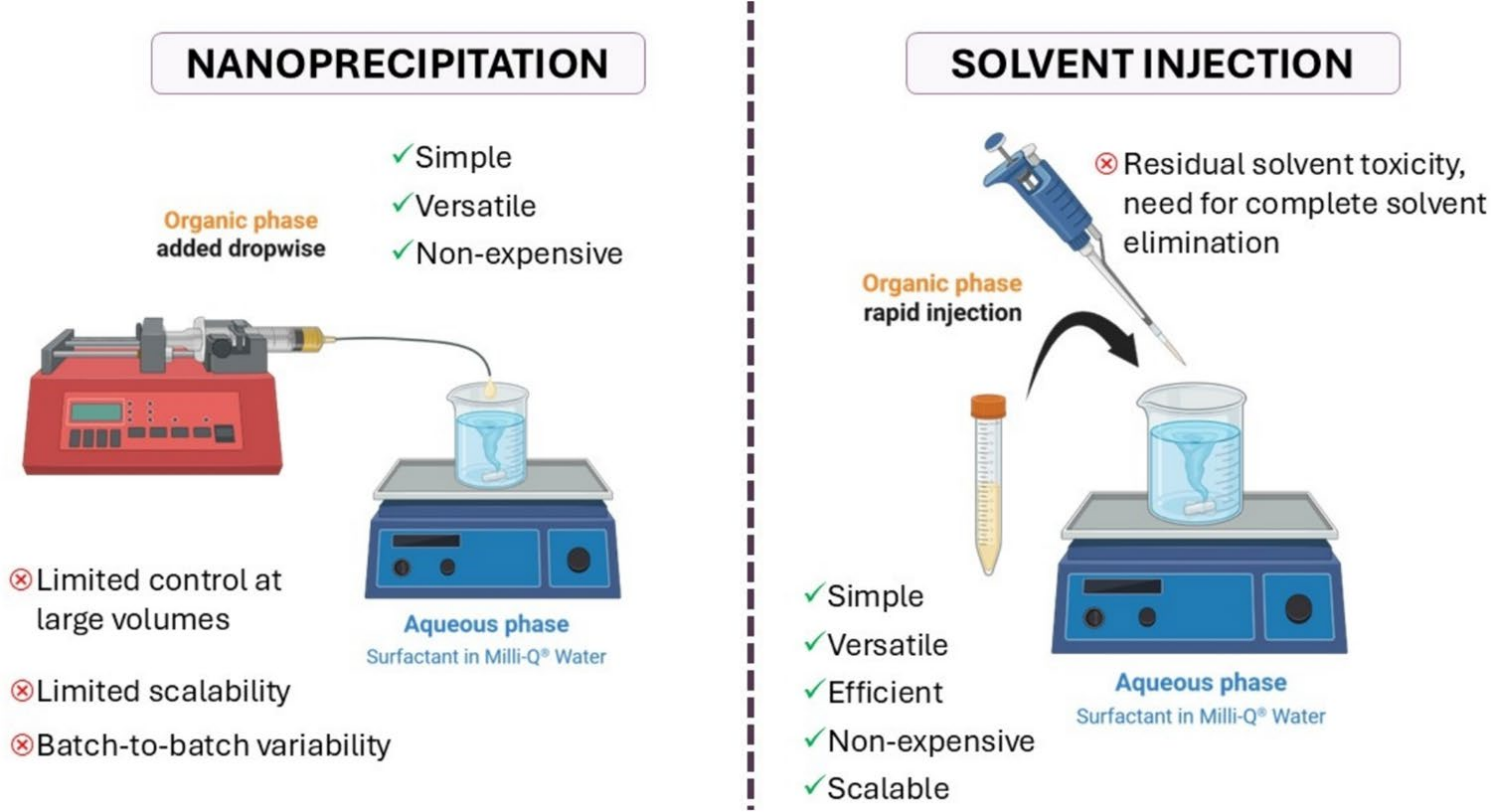
Schematic representation of nanoprecipitation and solvent injection methods with their respective advantages (green tick) and disadvantages (red cross)

Nanoprecipitation is also known as solvent displacement and leads to spontaneous emulsification [65]. Briefly, an organic phase (OP) is added dropwise to an aqueous phase (AP) while this one is continuously stirred. The OP contains lipids and hydrophobic drugs dissolved in a water-miscible solvent, and the AP contains a surfactant. The addition is usually performed using an injection pump, which can control the rate flow injection of the OP into de AP. Rapid desolvation (i.e., the removal of solvent molecules from lipids and drugs) drives the precipitation of LBNPs and facilitates efficient drug encapsulation (Fig. 10) [73].

Size and encapsulation efficiency can be tuned by adjusting variables such as stirring rate, solvent ratio, and the concentration of lipids, surfactants, and drugs. Among these parameters, mixing time is particularly critical for achieving smaller NPs, as it must be shorter than the characteristic precipitation time to ensure that mixing is completed before NPs formation begins. However, this method offers limited control over fluid dynamics, especially at large volumes, often resulting in broader size distributions. Incomplete or inefficient mixing not only affects uniformity but also contributes to batch-to-batch variability, ultimately limiting the scalability and reproducibility of the process (Fig. 10) [73].

#### Solvent injection

Solvent injection is a formulation method that relies on the rapid mixing of a lipid-dissolved OP into an AP, typically in a turbulent manner. Unlike nanoprecipitation, where mixing may occur under milder conditions, solvent injection involves injecting a solution (commonly ethanol containing phospholipids and drugs) into a larger volume of water or buffer. Upon contact with the aqueous phase, phospholipids spontaneously organize into bilayer fragments that precipitate and later fuse to form vesicles. The morphology of the resulting vesicles can vary depending on parameters such as the volume and speed of solvent injection. For example, a slower injection of ethanol at concentrations below 7.5% v/v typically yields unilamellar vesicles, while faster injection results in multilamellar structures (Fig. 10) [72].

Solvent injection method has been widely explored for the preparation of LBNPs due to its simplicity, efficiency, and adaptability. It does not require complex equipment, making it suitable for rapid and scalable production using pharmaceutically acceptable solvents [74]. Process parameters such as solvent type, lipid concentration, injection rate, and viscosity can be tuned to control particle size. A key factor in NPs formation is the diffusion of the solvent from the OP into the AP, although the exact mechanisms remain partially understood [74]. Despite its advantages, the technique presents notable limitations. The use of organic solvents, although generally regarded as safe, may cause toxicity concerns depending on the route of administration [74]. Moreover, incomplete removal of residual solvents can affect product safety and performance [72]. Thus, strategies for solvent elimination such as evaporation, freeze-drying, ultrafiltration, or washing are crucial steps that warrant further investigation (Fig. 10) [74].

#### Solvent-based emulsification

**Fig. 11:**
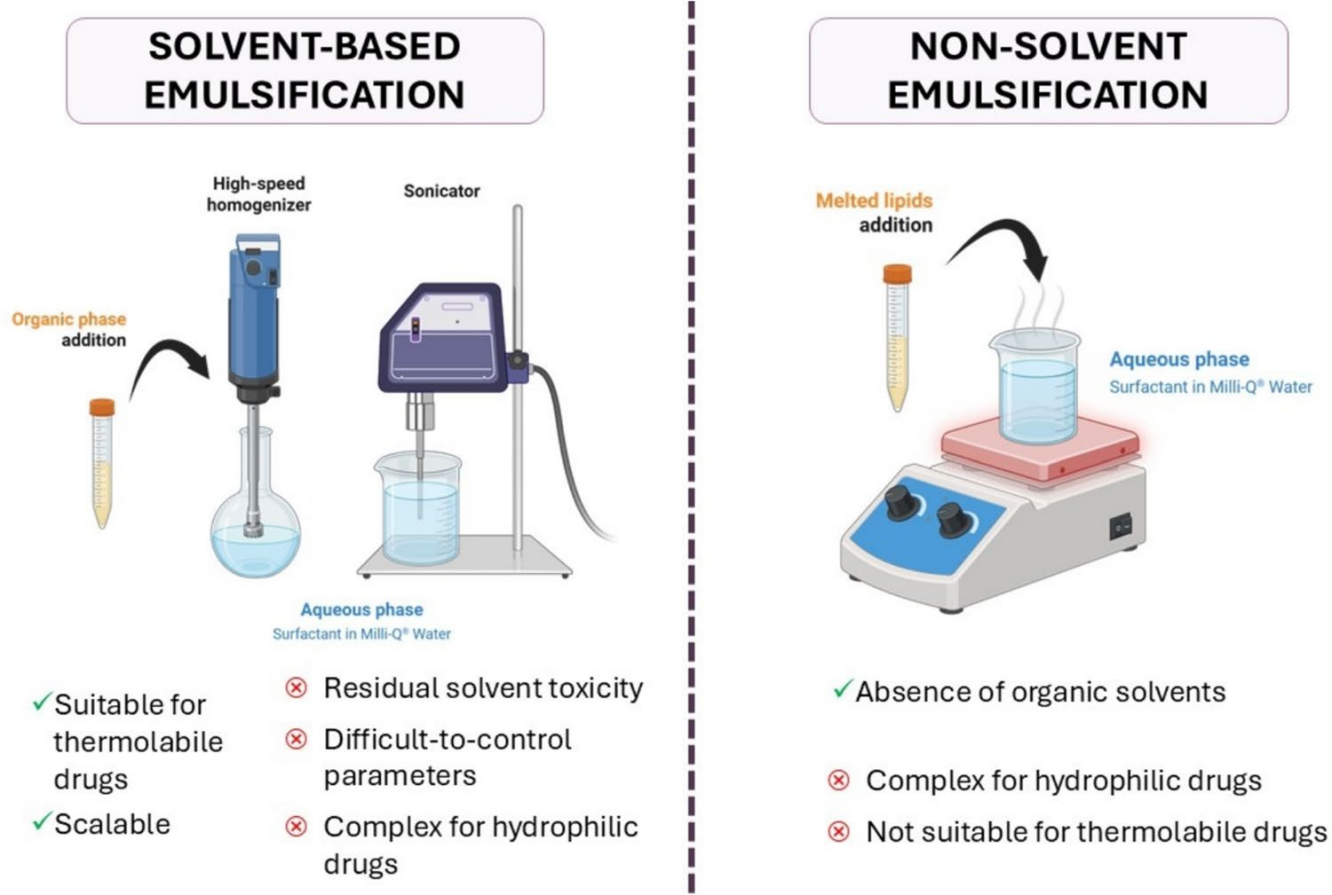
Schematic representation of solvent-based emulsification and non-solvent emulsification methods with their respective advantages (green tick) and disadvantages (red cross)

The solvent-based emulsification method is a widely used technique for the production of LBNPs. In this approach, lipids (solid or liquid) and hydrophobic drugs are first dissolved in a water-immiscible organic solvent, such as chloroform, cyclohexane, or toluene. This OP is then emulsified into an AP to form an oil-in-water emulsion (Fig. 11). Upon evaporation of the organic solvent, LBNPs precipitate and remain suspended in the aqueous medium [64, 73]. Variations of this method include the solvent emulsification-diffusion technique, in which a partially water-miscible solvent (e.g., benzyl alcohol or butyl lactate) is saturated with water to reach thermodynamic equilibrium. The lipid is dissolved in this water-saturated solvent and emulsified with a surfactant-containing AP. Rapid dilution and stirring lead to solvent diffusion and lipid precipitation, resulting in NPs formation [68].

This method offers notable advantages, including its suitability for encapsulating temperature-sensitive drugs and its compatibility with continuous, scalable production processes [68, 73]. Particle size can be influenced by factors such as lipid concentration and emulsion parameters [64]. However, this technique also presents several limitations. The use of organic solvents raises concerns regarding residual toxicity. Additionally, the process may involve difficult-to-control parameters that impact NPs size distribution [73]. Moreover, drug encapsulation depends on drug solubility: hydrophobic drugs are typically loaded using a single emulsion step, whereas hydrophilic drugs require a more complex double-emulsion approach (Fig. 11) [68].

#### Non-solvent emulsification

Non-solvent emulsification, also known as melting emulsification method, is an alternative technique to solvent-based emulsification for producing SLNs. Rather than dissolving lipids in organic solvents, this method involves melting solid lipids at temperatures 5–10 °C above their melting point and subsequently emulsifying them in an aqueous surfactant solution. The resulting oil-in-water emulsion can be processed using various techniques, including high-pressure homogenization, high-speed stirring, or ultrasonication (Fig. 11). Upon cooling, typically in an ice bath, the lipid phase solidifies, resulting in the formation of SLNs. Homogenization and sonication time, lipid and surfactant concentration, drug concentration, and the physicochemical properties of both lipids and surfactants, are formulation parameters that significantly influence NPs characteristics [73].

A key advantage of non-solvent emulsification methods is the absence of organic solvents, which eliminates the risk of solvent-related toxicity in the final LBNPs suspension. This makes the method particularly attractive for pharmaceutical applications requiring high biocompatibility. However, its efficiency is limited by the solubility of the drug in the lipid phase. Moreover, the high temperatures required to melt the lipids may compromise the chemical stability of thermolabile drugs (Fig. 11). Therefore, while this method offers a safer solvent-free approach, its applicability may be restricted by the physicochemical characteristics of both the drug and lipid components [73].

#### Thin film hydration

**Fig. 12:**
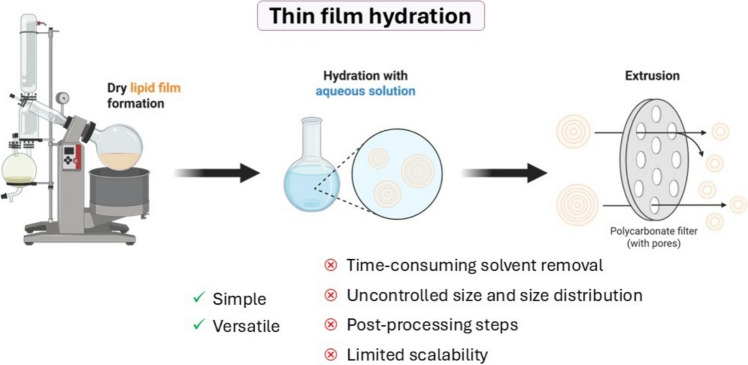
Schematic representation of thin film hydration method with its respective advantages (green tick) and disadvantages (red cross)

The thin film hydration method, also known as the Bangham method, is the earliest and the most common employed technique for liposome formulation. Briefly, lipids and amphiphilic molecules are dissolved in an organic solvent (commonly chloroform, ethanol, or methanol) and the solution is subsequently evaporated under vacuum using a rotary evaporator. This results in the formation of a thin, dry lipid film on the wall of a round-bottom flask [69, 72]. The film is then hydrated with an aqueous or buffer solution, often containing hydrophilic drugs, at a temperature above the lipid's phase transition temperature, which facilitates the formation of multilamellar vesicles [69]. The characteristics of the resulting liposomes are influenced by several parameters, including the volume and rate of hydration, lipid composition, and the temperature of the hydration medium. To obtain unilamellar vesicles of defined size, the suspension can be processed by sonication or extrusion through membranes with defined pore sizes (Fig. 12) [69].

Despite its simplicity and versatility, the thin film hydration method presents several limitations. The removal of organic solvent is time-consuming, often requiring extended vacuum application [72]. Additionally, the technique inherently lacks precise modulation of liposome size and size distribution, necessitating post-processing steps such as sonication or extrusion to achieve nanoscale vesicles [69, 72]. While sonication is convenient and suitable for processing large volumes, it may lead to lipid degradation, reduced encapsulation efficiency, and less uniform particle populations compared to extrusion [69]. Moreover, the method operates in batch mode and is difficult to scale up. Nevertheless, its ease of preparation, compatibility with a wide range of lipid compositions, and potential for encapsulating both hydrophilic and lipophilic drugs make it a valuable technique for laboratory-scale liposome production (Fig. 12) [64].

#### Microfluidics

Microfluidic technology is an advanced method for the controlled production of LBNPs through precise fluid manipulation in microchannels of ~ 150 × 300 µm [72]. Unlike conventional methods, microfluidics enables rapid and uniform mixing of the OP and AP, often using hydrodynamic flow focusing or micromixer geometries. These systems generate narrow, focused fluid streams that promote efficient mixing by diffusion within milliseconds. Microfluidic devices are generally categorized into chip-based and capillary-based platforms. Chip-based devices are fabricated using techniques like lithography or micromilling, allowing custom microchannel designs for homogeneous LBNPs production. Capillary-based systems, often made from glass, consist of coaxially aligned capillaries through which the OP and AP are combined to form NPs. This method enables the preparation of a variety of NPs, including SLNs and siRNA-loaded LBNPs [73].

The use of microfluidics offers several significant advantages over conventional methods. These include enhanced tuning of particle size and polydispersity, high encapsulation efficiency and excellent reproducibility. The process is also scalable and allows for continuous production, making it suitable for both research and industrial applications. However, chip-based devices are expensive and time-consuming to fabricate, and their performance may be affected by factors such as material compatibility, surface properties, and channel fouling. Capillary-based systems, while more cost-effective and chemically robust, pose challenges in terms of precise alignment and device assembly [73]. Additionally, microfluidic methods often result in low final concentration of NPs suspensions, requiring downstream concentration steps, and may be susceptible to channel clogging [72]. Despite these challenges, microfluidics remains a highly promising platform for the reproducible and tunable synthesis of LBNPs.

#### Supercritical fluid technology

Supercritical fluid (SCF) technology is an advanced method for NPs production that utilizes CO₂ under supercritical conditions (scCO₂), where it exhibits both gas- and liquid-like properties. In this process, lipids and drugs are typically dissolved in an organic solvent and subsequently exposed to scCO₂, forming a gas-expanded liquid. Various approaches have been developed based on this principle. Briefly, scCO₂ acts as a solvent whose capacity to dissolve solid lipids and drugs increases under high-pressure conditions. When the mixture is subjected to rapid depressurization, the system becomes supersaturated, leading to the precipitation of drug-loaded LBNPs [73]. Recently, SuperSomes process has been introduced, involving continuous mixing of ethanolic lipid solutions with scCO₂ and subsequent injection into an aqueous phase containing hydrophilic drugs, forming inverted micelles that reorganize into vesicles [72].

SCF-based methods offer notable advantages for LBNPs formulation. These include controlled particle size, narrow size distribution, high encapsulation efficiency (often above 90%), and complete removal of organic solvents. Additionally, the technique operates in continuous mode and allows solvent recycling, contributing to scalability and industrial feasibility [72]. However, one of the main drawbacks is the high cost and complexity of SCF equipment, which may hinder large-scale production. Furthermore, despite the tunable solvent power of scCO₂, the solubility of many drugs in this medium remains limited, necessitating the use of cosolvents or specific formulations. Finally, device design and precise computational modeling are essential to ensure reproducibility and performance, highlighting the high operational requirements of SCF-based systems [73].

## Clinical translation and rational engineering of LBNPs

### Clinically approved lipid-based nanomedicines

Among LBNPS, only liposomes have received FDA approval for cancer treatments (Table 2). ***Doxil®***, a PEGylated liposomal Doxorubicin, was the first FDA-approved nanomedicine in 1995 for solid tumors like breast and ovarian cancer, offering lower cardiotoxicity, prolonged circulation, and enhanced tumor targeting via the EPR effect [27]. In 1996, the EMA approved the use of ***Caelyx®*** as the European equivalent of Doxil®.

***DaunoXome®*** is a non-PEGylated liposomal formulation for the encapsulation of Daunorubicin, with increased drug delivery and reduced toxicity. In 1996, the FDA approved its use for the treatment of Kaposi’s Sarcoma [22]. Non-PEGylated liposomal Doxorubicin, marketed under the brand name of Myocet®, was approved by the EMA in 2000 for the treatment of breast cancer. Due to the absence of PEGylation, Myocet® is rapidly uptake by macrophages, resulting in shorter circulation time and limited tumor-targeting capacity compared to PEGylated liposomal formulations [27]. Immunostimulatory effects and prolonged plasma half-life of Mifamurtide were achieved through its non-PEGylated liposomal encapsulation (***Mepact®***) [22]. To date, Mepact® remains the only nanomedicine approved specifically for osteosarcoma, having received EMA authorization in 2009. Non-PEGylated liposomal Vincristine (***Marqibo®***) was approved against acute lymphoblastic leukemia in 2012 by the FDA. This formulation improves the delivery of Vincristin to the tumor site, decreases its systemic toxicity, enhances its solubilization and allows for sustained release [22].

***Onivyde®***, a PEGylated liposomal formulation of Irinotecan, was approved by the FDA in 2015 for the treatment of advanced pancreatic cancer, followed by EMA approval in 2016 [75]. This liposomal encapsulation enhances the PK profile of Irinotecan by increasing its plasma half-life, as liposomes protect the drug from premature metabolic degradation [76].

***Vyxeos®*** constitutes a pioneering and prominent clinical evidence for multidrug delivery. This dual-drug liposome coencapsulates Daunorubicin and Cytarabine in a fixed 1:5 ratio, which has shown synergic effect killing leukemia cells. Vyxeos® represents the most recent example of LBNPs approval, in 2017 by the FDA and in 2018 by the EMA [29].

### Administration routes

Composition and physicochemical properties of NPs can be modified to adapt them to numerous applications and routes of administration. Although systemic administration is the most common route for NPs applications, it often results in widespread distribution and lacks the desired targeting and specificity. In contrast, local delivery methods may improve targeting by allowing NPs to bypass some of the barriers associated with systemic delivery. However, these methods typically require more invasive procedures and complex techniques, which have their limitations. Moreover, local delivery is generally limited to diseases where the pathology is confined to known, accessible sites, such as certain solid tumors or traumatic injuries [77]. Optimizing the administration route can significantly influence biodistribution. For example, polymeric PLGA NPs accumulate primarily in the liver and spleen when administered intravenously, whereas subcutaneous or intranodal injection results in greater accumulation in local lymph nodes, which may be beneficial in certain immunotherapeutic applications [78].

#### Oral

Oral administration is the most used route in clinical practice and the one most preferred by patients. Its advantages include being non-invasive and not requiring medical supervision. However, the gastrointestinal tract (GIT) poses several biological barriers that can significantly reduce the bioavailability of many drugs, particularly those that are poorly soluble or unstable in digestive conditions [64]. In recent years, Nanomedicine has proposed various oral DDS to improve drug stability and absorption through the GIT. LBNPs have shown promise in this context. Their small size and large surface area allow for better interaction with the intestinal mucosa, while their composition can be optimized to resist enzymatic degradation and variable pH. For instance, PEGylation has been shown to protect LBNPs from degradation and aggregation, improving their performance in oral delivery [79]. Eudragit®, a polymeric coating (methyl acrylate, methyl methacrylate and methacrylic acid) designed to stabilize oral formulations, constitutes another example of a strategy to improve the stability of LBNPs for oral drug delivery [26].

Despite these advances, the complexity of the GIT and the variability between patients still represent major challenges. Nevertheless, LBNPs remain one of the most promising strategies for improving oral bioavailability and achieving effective systemic delivery through this route.

#### Parenteral

Intravenous administration is the most common route for chemotherapeutic drugs, especially when oral delivery is not possible due to the enzymatic degradation of compounds like peptides and proteins in the GIT. In these cases, repeated parenteral dosing is often required. LBNPs have shown great potential for parenteral drug delivery, as they can provide controlled release over extended periods. Their small size allows them to circulate easily through the microvascular system and improve therapeutic efficacy. When coated with PEG, NPs bioavailability is enhanced by reducing phagocytic uptake [64]. Several NPs formulations already approved for clinical use, such as Onpattro®, Vyxeos®, and Hensify®, are administered either intravenously or directly into tumors [77].

Compared to oral formulations, parenteral NPs require stricter control over particle size and polydispersity, as well as sterile production methods. They also need to meet specific criteria, such as serum stability and compatibility with blood components, to be safe and effective for intravenous use [68].

#### Alternative routes

***Rectal administration*** is an alternative route within the digestive system, though less frequently used than oral delivery due to lower patient compliance. Its physiological characteristics, such as low enzymatic activity and the absence of villi and microvilli, result in a reduced absorption surface but contribute to improved drug stability. In addition, the lower region of the rectum drains into the inferior vena cava and lymphatic vessels, bypassing hepatic metabolism [65]. This route is commonly used in pediatric treatments for its ease of administration and it is particularly suitable in situations requiring rapid drug absorption, where it can achieve higher plasma levels and therapeutic efficacy than oral administration at the same dose [64]. Recent innovations in rectal delivery, such as thermosensitive gel loaded with drug-encapsulated transferosomes, have successfully demonstrated enhanced bioavailability and significant antitumor efficacy against colorectal cancer [80].

The respiratory system offers a non-invasive route for both local and systemic drug delivery through ***nasal and pulmonary administration***. Pulmonary delivery benefits from the large surface area of the alveolar region, high vascularization, and limited first-pass metabolism, enabling rapid systemic absorption. In contrast, nasal delivery is particularly useful for targeting the brain, as it allows drugs to bypass the blood–brain barrier (BBB) via the olfactory pathway [65]. The lipid composition of LBNPs facilitates their passage across the BBB, and intranasal administration has shown greater efficacy in brain delivery compared to other routes [68]. However, mucus clearance, surfactants, and macrophages in the respiratory tract represent significant challenges to NPs stability and absorption. The formulation of inhaled drugs requires precise NPs design to ensure deposition at the targeted site [65]. For labile drugs degraded in the GIT, nasal delivery offers a viable alternative [64]. Recent studies have explored the potential of LBNPs in pulmonary cancer therapy by utilizing intratracheal liposomes for metastatic lung cancer, or by formulating SLNs that achieve high lung retention and enhanced tumor inhibition [81, 82].

***Ocular drug delivery*** is predominantly used for local treatment via eyedrops due to the anatomical and physiological barriers that hinder effective drug penetration. The eye's protective mechanisms, such as tear production, continuously clear the ocular surface, thereby diluting administered drugs and reducing their residence time. Additionally, structural barriers like the cornea limit drug absorption: the corneal epithelium restricts the passage of hydrophilic and ionic compounds, while the hydrophilic stroma impedes lipophilic drugs. Although this reduces systemic absorption, it also creates a drug reservoir effect that can support sustained release [65]. To improve ocular bioavailability, DDS such as PEG-coated polymeric NPs and SLNs have been explored [64]. LBNPs are particularly promising due to their small size, which avoids visual obstruction, and their mucoadhesive properties, which reduce clearance by ocular defenses [68]. Ocusolin™, an AlphaRx Inc. developed product, is based on SLNs for Gentamicin delivery and has demonstrated its potential for ocular infections in preclinical studies [64]. Focusing on cancer, SLNs have been used to encapsulate Etoposide, achieving sustained drug release and prolonged vitreous retention following a single intravitreal injection, highlighting their potential for safer and more effective ocular delivery targeting diseases such as retinoblastoma [83].

The skin is structurally composed of three distinct layers. (1) The epidermis, an avascular outer layer, serves as a biological barrier against microbial invasion and plays a critical role in maintaining homeostasis. Beneath it, (2) the dermis provides thermal protection through its dense vascular network, while (3) the hypodermis offers mechanical support. Although the skin is primarily employed for ***topical drug administration***, certain compounds are capable of penetrating its barrier to reach systemic circulation. This transdermal route bypass first-pass hepatic metabolism and allows for sustained plasma drug concentrations [65]. LBNPs, owing to their favorable physicochemical properties and biocompatibility, are particularly suitable for topical delivery. Their nonirritant and nontoxic lipid matrix offers significant advantages over alternative delivery systems [64]. Accordingly, different LBNPs have been explored for the topical administration of active pharmaceutical ingredients. Encapsulating Rose Bengal, a sono-photosensitizer drug, in transferosomes facilitated deep skin penetration and protected the payload from photodegradation, thereby improving its antimelanoma efficacy without the need for light activation [84].

### Therapy-matched LBNPs engineering

To successfully overcome the specific PK limitations and biological barriers of each targeted therapy, LBNPs must be rationally engineered rather than applying a one-size-fits-all approach. By integrating the current knowledge on administration routes, formulation methods, and surface modifications, we can establish a therapy-matched design framework. Table 3 provides a comprehensive synthesis of the dominant barriers for mAbs, ADCs, siRNA, SMIs, and PROTACs, mapping them to specific LBNP design recommendations.

**Table 3:** Therapy-matched LBNP design: overcoming biological barriers of targeted therapies

Targeted therapy	Key clinical limitations	Dominant biological barriers	Design recommendations & strategies
mAbs	Poor tumor penetration Intravenous-only administration Immunogenicity	Limited extravasation Dense tumor stroma	**Design** Conjugation to LBNP surface (ACNPs) Use of antibody fragments Optimized PEGylation density **Formulation** Mild methods
ADCs	Linker instability Limited internalization Low efficiency On-target, off-tumor toxicity	Systemic circulation (premature payload release) Heterogeneous penetration	**Design** ACNPs to bypass linkers and increase payload capacity SLNs for stability **Formulation** Mild methods
siRNA	Nuclease degradation Rapid clearance Immunogenicity Off-target effects	Poor cellular permeability Frequent endosomal entrapment	**Design** LNPs engineered with ionizable lipids Stealth coating **Formulation** Microfluidics
SMIs	Low oral bioavailability Poor aqueous solubility Rapid resistance Short half-life	Biological fluids (solubility issues) Gastrointestinal tract barriers (for oral administration)	**Design** NLCs and surfactants to maximize payload solubility Optimized for alternative routes (e.g., oral, respiratory) **Formulation** Non-solvent emulsification (avoids organic solvent toxicity) Supercritical fluid technology for high encapsulation of hydrophobic drugs
PROTACs	Large molecular weight (> 1000 Da) Dose-limiting "Hook effect” Suboptimal solubility	Poor membrane permeability Endosomal sequestration	**Design** pH-responsive systems Lipid optimization for endosomal escape Targeted delivery to optimize local dose **Formulation** Advanced continuous methods to ensure high encapsulation efficiency and reproducibility for large, complex hydrophobic molecules

*mAbs* Monoclonal antibodies, *ADCs* Antibody–drug conjugates, *siRNA* Small interfering RNA, *SMIs* Small molecule inhibitors, *PROTACs* PROteolysis TArgeting Chimeras, *LBNPs* Lipid-Based Nanoparticles, *ACNPs* Antibody-Conjugated Nanoparticles, *SLNs* Solid Lipid Nanoparticles, *LNPs* Lipid Nanoparticles, *NLCs* Nanostructured Lipid Carriers

As detailed in Table 3, the structural complexity and specific biological barriers of main targeted therapies dictate distinct engineering and manufacturing requirements. For macromolecules such as mAbs and ADCs, classic encapsulation within the LBNP core is often inefficient and technically challenging [85, 86]. Instead, strategically conjugating these targeting moieties to the LBNP surface to form ACNPs circumvents issues related to linker instability and payload capacity [21, 45, 46]. To overcome the poor tumor penetration characteristic of full-sized antibodies, substituting them with smaller engineered antibody fragments, such as scFv or Fab, significantly enhances extravasation and cellular internalization [21, 48]. Furthermore, navigating the "PEG dilemma" by precisely tuning the density of stealth coatings is critical for these systems to balance prolonged circulation with effective tumor penetration [56]. From a manufacturing perspective, the thermolabile nature of these biologic proteins necessitates mild formulation conditions, making solvent-based emulsification highly suitable compared to melting techniques [68, 73].

For nucleic acid-based therapies like siRNA, the primary engineering challenge shifts from surface conjugation to overcoming intracellular barriers and avoiding rapid clearance. The obligatory strategy relies on formulating LNPs with ionizable lipids to actively facilitate endosomal escape, alongside stealth coatings to prevent nuclease degradation [9, 59, 87, 88]. Because these multi-component systems require precise electrostatic complexation, microfluidic platforms are the formulation method of choice to ensure reproducible, continuous-flow synthesis with narrow size distributions [72, 73].

Finally, the translation of hydrophobic molecules, including SMIs and PROTACs, is primarily dictated by solubility and pharmacokinetic constraints. For SMIs, formulating solid matrices like SLNs or NLCs maximizes payload solubility and stability, enabling their adaptation for alternative administration routes (e.g., oral or respiratory) [65, 68]. These specific lipid carriers can be optimally produced via non-solvent emulsification to avoid organic solvent toxicity [73]. PROTACs, meanwhile, face unique delivery limitations due to their high molecular weight and the "Hook effect", necessitating highly controlled, targeted, or pH-responsive release systems to optimize local dosing. To achieve high encapsulation efficiencies for these complex molecules, advanced techniques like supercritical fluid technology overcome the drawbacks of conventional batch processing [72, 73]. Translating these sophisticated, therapy-matched designs from the bench to bedside, however, introduces a new set of manufacturing and regulatory hurdles.

### Translational challenges of LBNPs

While several LBNPs have reached clinical approval (Table 2), their translation faces well-documented hurdles in chemistry, manufacturing, and controls (CMC), scale-up, reproducibility, regulatory pathways, and cost-effectiveness barriers. Liu et al. provide a recent systematic analysis, revealing that ~ 79% of nanomedicines fail phase III primarily due to undervalue clinical efficacy despite promising preclinical data [89].

***CMC and scale-up limitations*** remain critical, as laboratory-scale methods (thin-film hydration, solvent injection) struggle to maintain batch-to-batch consistency of particle size, lipid composition, and drug loading during industrial scaling [90–92]. Microfluidic platforms and supercritical fluid technologies offer solutions but require extensive process analytical technology validation under GMP conditions [93, 94].

***Regulatory pathways*** follow conventional drug frameworks at FDA/EMA. As explicitly stated in EMA's January 2025 Horizon Scanning Report, “There is no dedicated EU legal framework for nanomedicines; they are regulated under existing medicines legislation”. This conventional regulatory pathway is supplemented by EMA reflection papers addressing specific platforms (liposomes, block copolymer micelles, surface coatings, intravenous iron nanoparticles), but lacks comprehensive nano-specific legislation. This highlights the necessity of harmonized analytical methods and reference standards [90, 95].

***Failures and withdrawals*** illustrate different translational gaps. DepoCyt®, a liposomal cytarabine approved in 1999, showed improved response rates and survival versus conventional cytarabine in lymphoma and leukemia, yet its development was ultimately discontinued in 2018 due to CMC problems that could not be resolved to GMP standards, highlighting how technical production issues can terminate an otherwise active product [96–98]. Apealea®, a paclitaxel micellar formulation approved by EMA in 2018 for ovarian cancer, achieved only marginal absolute benefit, and its subsequent commercial discontinuation in 2024 underscores how modest efficacy gains and pricing pressures limit the real-world viability of many cancer nanomedicines [99, 100]. Beyond outright withdrawals, some nanomedicine programs have failed at the regulatory stage despite apparently positive clinical data: Opaxio® (paclitaxel poliglumex), for instance, showed a 42‑day overall‑survival gain yet its European application was withdrawn after concerns about comparator selection, endpoint selection and trial regimen, underscoring that efficacy signals alone are insufficient without a well‑justified study design [101]. In contrast, successes such as Vyxeos®, suggest that nanomedicines backed by a clear mechanistic rationale, robust PK/PD justification and rigorously designed pivotal trials are better positioned to overcome regulatory and market hurdles [96, 102].

## Conclusions

The clinical efficacy of targeted anticancer therapies—including mAbs, ADCs, siRNA, SMIs, and PROTACs—is frequently compromised by PK and PD barriers. LBNPs offer a highly versatile, biocompatible, and rationally engineered platform to overcome these systemic limitations. The current preclinical and clinical landscapes of nanomedicine yield several critical take-home messages:

- *Therapy-matched design is essential*. There is no universal LBNP formulation. Successful design must be rationally tailored to the specific biological barrier of the payload. This includes utilizing ionizable lipids for the endosomal escape of nucleic acids, solid lipids for the stabilization of ADC linkers, or advanced NLCs to improve the solubility of hydrophobic SMIs.
- *Moving beyond the EPR effect****.*** While passive targeting via the EPR effect remains the foundational mechanism for clinically approved nanomedicines, its high inter- and intra-tumoral heterogeneity limits its reliability as a universal strategy. The integration of active targeting, immune-modulating combinations, and alternative delivery strategies is necessary to achieve consistent clinical benefits in poorly perfused tumors.
- *Translational bottlenecks dictate clinical success*. The transition from bench to bedside is currently hindered less by a lack of preclinical efficacy, and more by CMC challenges, scalability issues, and the absence of specific regulatory frameworks. Clinical success requires formulations backed by robust PK justification and technically viable manufacturing processes.

## Future perspectives: research gaps and priority directions

**Fig. 13:**
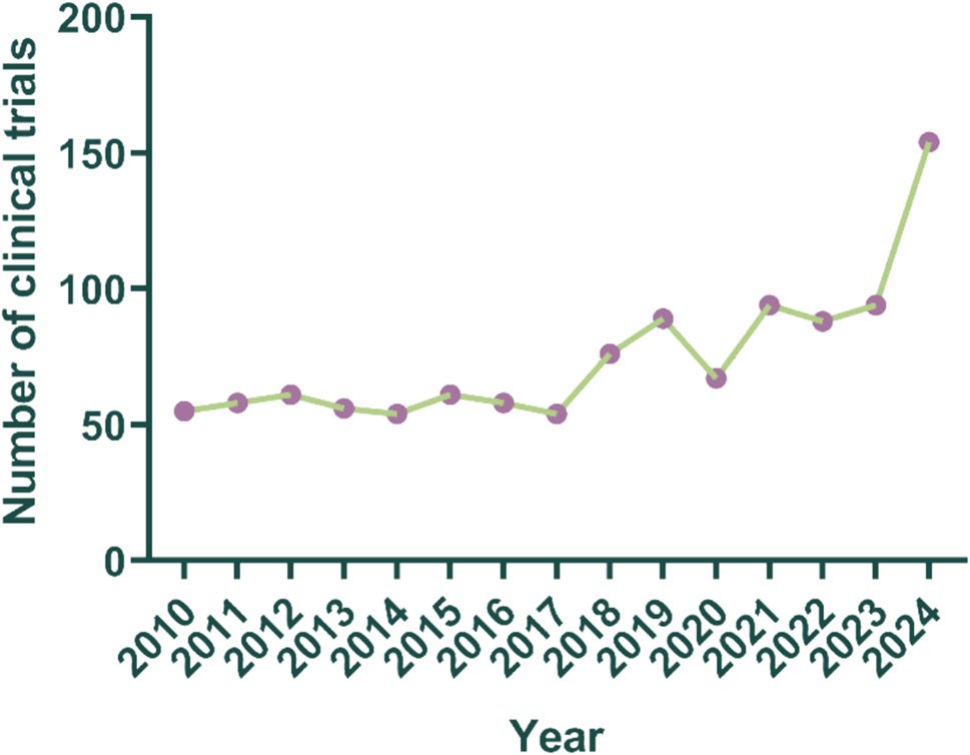
Number of cancer-related clinical trials involving nanomedicines registered per year (2010–2024). Data were retrieved from ClinicalTrials.gov using the Expert Search tool with the following query: (cancer OR tumor OR neoplasm OR malignancy) AND (liposome OR lipid nanoparticle). Only interventional studies with a start date between January 1, 2010, and January 1, 2025, were included. Data accessed on June 2025

As previously discussed, clinicals findings highlight the versatility and therapeutic potential of nanomedicine in cancer treatment. The growing interest in the development of novel nanomedicines for clinical use is reflected in Fig. 13. Over the past 15 years, more than 1,100 clinical trials have been conducted involving nanomedicine-based strategies for cancer therapy. The number of such trials has progressively increased over time. During the COVID-19 pandemic, the number of clinical trials declined; however, in 2021, a rebound was observed, and trial activity began to increase once again. The successful use of COVID-19 vaccines represented a turning point for LBNPs. A significant rise was observed in 2024, underscoring the up-to-date nature of this research field.

Despite this recent resurgence and clinical validation of LBNPs, several key research gaps must be addressed to accelerate the translation of next-generation oncological nanomedicines. The priority directions for future research include:

- *Elucidating endosomal escape mechanisms*. Although endosomal escape is frequently highlighted in the context of nucleic acid-based therapies [59, 87, 103], it also represents a critical translational barrier for other targeted therapies, such as PROTACs and certain SMIs. These drugs, when delivered via nanocarriers, must overcome endosomal sequestration to reach their intracellular targets and achieve therapeutic effect. Consequently, numerous studies have been conducted to elucidate the mechanisms underlying endosomal escape; however, no clear consensus has yet been reached [88, 104–106]. Priority should be given to developing quantitative mechanistic tools and systematic structure–activity relationships to design lipids and helper components that predictably facilitate endosomal escape.
- *Structural optimization and nomenclature consensus*. SLNs have emerged as promising systems to address limitations associated with other LBNPs by enhancing physical stability. However, uncontrolled drug expulsion during storage remains a significant challenge. While NLCs were developed to improve SLN performance, there is still critical room for optimization in their design and functionality. Furthermore, the rapid expansion of formulation techniques has led to a wide diversity of hybrid LBNPs, resulting in a lack of consensus in their classification. For instance, mRNA COVID-19 vaccines are variably described as LNPs (amorphous) or SLNs depending on the source [68]. Establishing a standardized nomenclature and optimizing these solid/hybrid lipid cores are priority directions for future research.
- *Exploration of alternative administration routes*. To bypass the limitations of systemic intravenous delivery and the hostile environment of the GIT, priority should be given to developing LBNPs optimized for non-invasive, alternative routes. Specifically, respiratory (pulmonary and intranasal) routes hold significant potential for overcoming the BBB and improving local bioavailability, requiring further innovation in mucoadhesive and cell-penetrating surface engineering.
- *Improving targeted delivery*. Active targeting improves the accumulation of LBNPs in tumors with low EPR effect and increases their specificity toward cancer cells. Strategies to modify and functionalize the surface of NPs in order to enhance their selective accumulation in tumor tissues represent a key area of ongoing research. In this context, conjugation methods are being optimized to overcome common limitations associated with ligand attachment, such as steric hindrance and protein corona formation, which can compromise the bioactivity of targeted systems and lead to off-target effects [107–109]. Another crucial challenge in conjugation is ensuring that the attached ligands remain stable under physiologically harsh conditions [65]. Numerous studies have demonstrated that the EPR effect serves as a foundational principle for achieving active targeted delivery [28]. Future research must prioritize advanced conjugation methods and stealth coatings beyond PEG (such as zwitterionic polymers or biomimetic cell-membrane coatings) to balance circulation time, immune evasion, and target recognition.
- *Advanced manufacturing and regulatory standardization*. Traditional formulation methods (e.g., thin-film hydration) struggle with batch-to-batch consistency during industrial scale-up. A critical priority is the broader adoption and GMP validation of continuous, controllable manufacturing technologies, such as microfluidics and supercritical fluid technology. This robust manufacturing must be coupled with the development of harmonized analytical methods to satisfy conventional EMA/FDA regulatory pathways, which currently lack dedicated nano-specific legislation.

## Data Availability

No datasets were generated or analysed during the current study.

## Abbreviations

*ACNPs*: Antibody conjugated nanoparticles
*ADC*: Antibody-drug conjugate
*AP*: Aqueous phase
*BBB*: Blood-brain barrier
*CMC*: Chemistry, manufacturing and controls
*DDS*: Drug delivery system
*EMA*: European Medicines Agency
*EPR*: Enhanced permeability and retention
*FDA*: Food and Drug Administration
*GIT*: Gastrointestinal tract
*HNSCC*: Head and neck squamous cell carcinoma
*HR*: Hormone receptor
*LBNPs*: Lipid-based nanoparticles
*mAb*: Monoclonal antibody
*NLC*: Nanostructured lipid carrier
*NPs*: Nanoparticles
*NSCLC*: Non-small cell lung cancer
*OP*: Organic phase
*PD*: Pharmacodynamic
*PDLLA*: Poly-D,L-lactide
*PEG*: Poly(ethylene glycol)
*PK*: Pharmacokinetic
*PLGA*: Poly(lactic-co-glycolic acid)
*PROTAC*: PROteolysis TArgeting Chimera
*PSMA*: Prostate-specific membrane antigen
*SCF*: Supercritical fluid
*siRNA*: Small interfering RNA
*SLNs*: Solid-lipid nanoparticles
*SMI*: Small molecule inhibitor
*SMIK*: Small molecule protein kinase inhibitor
*TNBC*: Triple negative breast cancer
