# Correction to: Rational design of lipid-based nanoparticles for targeted anticancer therapies

**DOI:** 10.1007/s13346-026-02132-7

**Published:** 2026-04-23

**Authors:** María Arenas‑Moreira, Alberto Ocaña, Carlos Alonso-Moreno, Iván Bravo

**Affiliations:** 1 Departamento de Química Inorgánica, Orgánica y Bioquímica, Facultad de Farmacia‑Centro de Innovación en Química Avanzada (ORFEO‑CINQA), Unidad nanoDrug, Universidad de Castilla-La Mancha, Albacete, 02008 Spain; 2 Experimental Therapeutics in Cancer Unit, Instituto de Investigación Sanitaria San Carlos (IdISSC), Madrid, Spain; 3 START Madrid‑Fundación Jiménez Díaz (FJD) Early Phase Program, Fundación Jiménez Díaz Hospital, Madrid, Spain; 4 Departamento de Química Física, Facultad de Farmacia, Unidad nanoDrug, Universidad de Castilla-La Mancha, Albacete, 02008 Spain

**Correction to: Drug Delivery and Translational Research**

10.1007/s13346-026-02110-z

In the original online version of this article, the graphical abstract was missing. In addition, there were errors in Figs. 1, 3, 6, 8, and 9. These errors occurred during the preparation of the final artwork and do not affect the scientific results, data interpretation, or the overall conclusions of the work. The original article was corrected.

